# Live-cell DNMT3A “catalysome” mapping using engineered methyl-transfer pathways

**DOI:** 10.1093/nar/gkag906

**Published:** 2026-09-22

**Authors:** Vaidotas Stankevičius, Liepa Gasiulė, Kotryna Kvederavičiūtė, Aleksandras Čečkauskas, Audronė Rukšėnaitė, Giedrė Urbanavičiūtė, Gražina Petraitytė, Viktoras Masevičius, Giedrius Vilkaitis, Saulius Klimašauskas

**Affiliations:** Institute of Biotechnology, Life Sciences Center, Vilnius University, 10257 Vilnius, Lithuania; Institute of Biotechnology, Life Sciences Center, Vilnius University, 10257 Vilnius, Lithuania; Institute of Biotechnology, Life Sciences Center, Vilnius University, 10257 Vilnius, Lithuania; Institute of Biotechnology, Life Sciences Center, Vilnius University, 10257 Vilnius, Lithuania; Institute of Biotechnology, Life Sciences Center, Vilnius University, 10257 Vilnius, Lithuania; Institute of Biotechnology, Life Sciences Center, Vilnius University, 10257 Vilnius, Lithuania; Institute of Biotechnology, Life Sciences Center, Vilnius University, 10257 Vilnius, Lithuania; Institute of Chemistry, Faculty of Chemistry and Geosciences, Vilnius University, 03225 Vilnius, Lithuania; Institute of Biotechnology, Life Sciences Center, Vilnius University, 10257 Vilnius, Lithuania; Institute of Chemistry, Faculty of Chemistry and Geosciences, Vilnius University, 03225 Vilnius, Lithuania; Institute of Biotechnology, Life Sciences Center, Vilnius University, 10257 Vilnius, Lithuania; Institute of Biotechnology, Life Sciences Center, Vilnius University, 10257 Vilnius, Lithuania

## Abstract

Catalytic interplay among three major DNA methyltransferases (DNMTs) establishes and maintains genomic modification patterns that define mammalian cell identity and transitions during development. Despite extensive molecular characterization, mechanisms and precise contributions of DNMT-specific methylation in native cellular contexts remain elusive. To address this, we employed structure-guided engineering of DNMT3A to enable catalytic transfer of extended azide tags from a synthetic cofactor analog, Ado-6-azide, onto DNA. By coupling orthogonal biocatalysis with pulse-internalization of Ado-6-azide or with metabolic conversion of the cofactor precursor, azido-methionine, we established two strategies for DNMT3A catalysis-dependent genome tagging in live mESCs under near-physiological conditions. Genome-wide mapping of DNMT3A catalysis (catalysome) in primed mouse embryonic stem cells facilitated discovery of opposing DNMT3A and TET dioxygenase activities at boundaries of CpG islands, promoter-proximal regions, and enhancers. DNMT3A also exhibited a strong enrichment at pericentric major satellites, reinforcing its key role in shaping the epigenetic centromere identity. Collectively with a complementary DNMT1-engineered cell line, we present a versatile platform for direct, selective, and time-resolved interrogation of individual DNA methylation writers in live cells under physiological conditions.

## Graphical Abstract

**Graphical Abstract:**
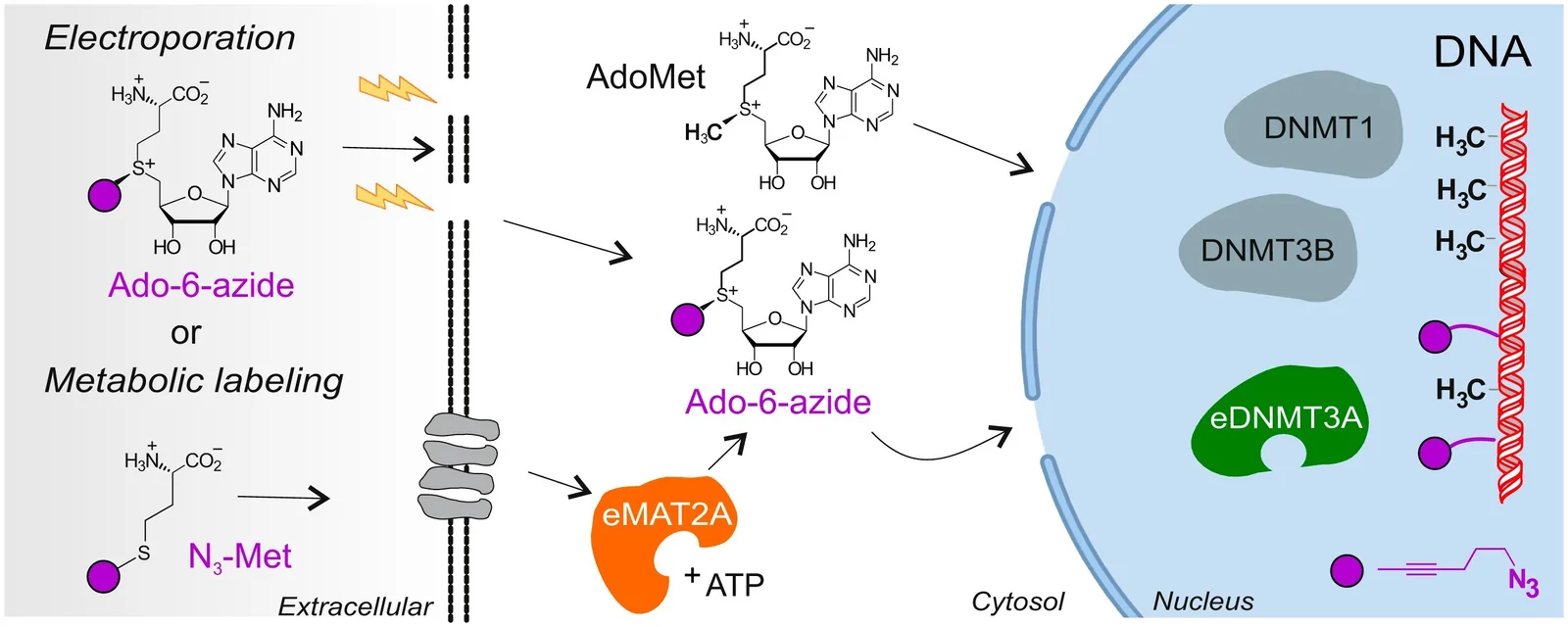

## Introduction

5-Methylcytosine (m^5^C) is a fundamental epigenetic DNA mark in mammalian genomes which facilitates vital cellular processes, including the maintenance of genome stability, the regulation of gene expression, and cell identity [1, 2]. Genomic methylation events are executed by the collaborative activity of DNA methyltransferase (DNMT) family enzymes [3, 4], which transfer a methyl group from the *S*-adenosyl-l-methionine (AdoMet) cofactor to the cytosines within CpG dinucleotides. DNA *de novo* methyltransferases DNMT3A and DNMT3B uphold the modification of unmethylated CpG cytosines, whereas DNMT1 maintains the inheritance of the DNA methylome in proliferating cells by preserving methylation patterns during DNA replication, thereby establishing lineage-specific methylation landscapes in differentiating cells during early embryogenesis and somatic cell differentiation. DNA methylation is a dynamic process that may be enzymatically eliminated by m^5^C dioxygenase TET family proteins [5–7], mostly by oxidative demethylation resulting in intricate and dynamic cellular methylomes. Conversely, an aberrant DNMT activity correlates with impaired cell function and subsequent developmental or somatic failures, frequently favoring the emergence of cancer cells [8–10], rendering them highly appealing targets for therapeutic applications [11].

Alongside the accepted classification of DNMTs into maintenance and *de novo* functions at the genomic level, numerous indications imply functional collaboration and competing activities of the methylation writers at specific genomic regions and/or developmental stages [12–16]. However, accurate determination of their functions remains problematic [17, 18], due to the lack of adequate techniques for following the dynamic methylation landscapes formed by each DNMT. The majority of currently available techniques focus on quantifying the cumulative m^5^C profiles, which arise from the combined activities of the three DNMTs and three TET dioxygenases. The catalytic activity of DNMTs is strongly modulated by post-translational modifications [19–21] and interactions with factors that gate chromatin accessibility [5, 22, 23], highlighting that spatial association with the protein co-localization alone may not indicate DNMT-specific activities in live cells. Moreover, perturbation of the DNMT protein levels triggers gradual genome-wide demethylation events that reshape the transcriptional and structural landscape of the epigenome [24–26] complicating efforts to extract the contributions of individual DNMTs in living cells.

A bump-and-hole strategy has emerged as a useful tool for dissecting complex multi-enzyme and multi-target systems [27]. In general, this technique introduces a “bump” (a steric extension) in the ligand and a compensatory “hole” (a structural accommodation) in the corresponding protein or enzyme, providing a tailored specificity of orthogonal protein–ligand or enzyme–substrate pairs for exclusive probing of genuine targets via designated activity. In the context of repurposing DNMT–AdoMet pairs, a novel Dnmt-TOP-seq approach was recently developed to monitor the catalytic activity of individual DNMTs in live mouse embryonic stem cells (mESCs) [28, 29]. It utilized a genetically engineered DNMT1 to transfer a 6-azidohex-2-ynyl group onto genomic DNA using a synthetic AdoMet cofactor analogue, Ado-6-azide. As AdoMet and its synthetic analogues cannot freely penetrate the cell membrane, Ado-6-azide was delivered into cells by increasing membrane permeability using electroporation. On the other hand, AdoMet is synthesized *in vivo* by methionine adenosyltransferase MAT2A using ATP and l-methionine (Met) as substrates [30], and thus similar bump-and-hole strategies has been applied for engineering of the MAT protein and corresponding Met derivates [31, 32]. Finally, installation, in mESCs, of a chromosomally expressed engineered MAT2A–DNMT1 cascade permitted metabolic in-cell production of Ado-6-azide from its synthetic precursor analogue, azide-6-methionine (N_3_-Met), and subsequent chemical tagging of DNMT1-specific methylomes under native conditions [33].

Taking advantage of comprehensive biochemical and structural characterization [34], and motivated by our successful repurposing of mouse DNMT1 [29], here we carried out a steric engineering of the catalytic pocket in mouse DNMT3A by replacement of R887 with a shorter alanine or glycine residue. We found that the DNMT3A R887G variant conferred efficient DNA azidoalkylation with Ado-6-azide in the presence of AdoMet *in vitro* and *in vivo*. Moreover, we observed a comparable accumulation of genomic azide tags and distribution of modified CpGs across a variety of genomic features in the respective CRISPR-edited *Dnmt3a* R887G mESC lines, when a synthetic cofactor or a cofactor precursor N_3_-Met was provided. Functional enrichment analysis indicated that DNMT3A R887G profiles were associated with prevalent modification of CpG islands, shores, and shelves, as well as promoter and gene body-associated genomic regions. Remarkably, the most substantial enrichment in DNMT3A targets was apparent in satellite sequences, particularly major satellites. Intriguingly, in contrast to previous findings, engineered DNMT3A exhibited no notable sequence preference in an octanucleotide window around the target CpG dinucleotide. Taken together with the previously reported DNMT1-engineered cell lines [29, 33, 35], we present a versatile platform for direct, selective and time-resolved interrogation of the *de novo* and maintenance DNA methylation writers.

## Materials and methods

### Synthetic cofactors

AdoMet analogue Ado-6-azide was chemically synthesized from AdoHcy as previously described [36]. For *in vivo* labeling, Ado-6-azide was utilized without chromatographic purification as a mixture of *R,S*- and *S,S*-diastereomers. Methionine derivative N_3_-Met (*S*-(6-azidohex-2-yn-1-yl)-L-homocysteine) was synthesized as previously described [33].

### Protein mutagenesis and isolation

The pPIC3.5K vector containing a His-tagged full-length mouse DNMTs methyltransferases DNMT3A WT, R887A and R887G were constructed as described previously [29]. Yeast transformants were established by electroporating SacI linearized plasmids into *Pichia pastoris* GS115 (his4) strain. His + transformants were selected on a minimal agar medium (0.67% YNB and 2% glucose) and clones with a high copy number of the respective *Dnmt3a* variant were subsequently picked on YPD-agar plates supplemented with 1 mg/ml geneticin (Sigma).

His-tagged methyltransferase protein variants were expressed in the yeast *P. pastoris* system. The yeast cells were cultivated in BMG medium (100 mM potassium phosphate, pH 6, 1.34% YNB, 4 × 10^−5^ % biotin, and 1% glycerol) at 30°C until OD_595_ of 2–2.5 was achieved, after which they were resuspended in BMM medium (100 mM potassium phosphate, pH 6, 1.34% YNB, and 4 × 10^−5^ % biotin) with a maximum of 3% methanol. Following 24 h of methanol induction, cells were collected and lysed using a Bead Beater (Roth) in a lysis buffer comprising 50 mM Na_2_HPO_4_, 10% sucrose, 250 mM NaCl, 3 mM MgCl_2_, 0.1% Triton X-100, 0.1 mM phenylmethylsulfonyl fluoride (PMSF), and cOmplete Mini EDTA-free Protease Inhibitor Cocktail (Roche), pH 7.4. The soluble fraction was obtained after centrifugation for 20 min at 45 000 × *g* and then filtered using a 0.45 µm Millex-HV PVDF filter (Millipore). The filtered lysate was applied to a HiTrap IMAC HP column (Merk) that had been preloaded with Ni^2+^ and equilibrated using wash buffer [50 mM Na_2_HPO_4_, 250 mM NaCl, 10% (w/v) sucrose, 3 mM MgCl_2_, 0.1% Triton X-100, and 5 mM 2-mercaptoethanol, at pH 6.2]. DNMT3A proteins were eluted using wash buffer containing 1 M imidazole (pH 6.2) and then dialyzed twice against buffer A overnight, followed by dialysis against the storage buffer (50 mM Na_2_HPO_4_, 25 mM NaCl, 0.1% Triton X-100, 5 mM 2-mercaptoethanol, and 50% glycerol, pH 7.4). Aliquoted proteins were stored at −20°C. MAT2A proteins were purified as described previously [33].

### Comparative analysis of the DNMT3A catalytic activity with AdoMet and Ado-6-azide

Catalytic reactions were carried out in reaction buffer (20 mM Tris–HCl, pH 7.4, 1 mM EDTA, 1 mM dithiothreitol (DTT), and 0.1 mg/ml bovine serum albumin) utilizing 800 nM DNMT3A protein variants, 6 μM DNA substrate (poly(dI-dC)·(dI-dC) or poly(dG-dC)·(dG-dC), unmethylated 25-mer oligonucleotide duplex 5′-ACAGTATCAGGCGCTGACCCACAAC/5′-GTTGTGGGTCAGCGCCTGATACTGT) and 100 μM cofactor, either alone or as a 1:1 premix of AdoMet and Ado-6-azide, for 4 h at 37°C. The enzyme was subsequently inactivated by heating for 10 min at 80°C. Samples were subjected to HPLC-MS/MS analysis as detailed below.

### Steady-state kinetics of DNMT3A variants

Steady-state turnover reactions were conducted in reaction buffer containing 50 nM DNMT3A variant, 100 μM AdoMet or Ado-6-azide and 3 μM DNA substrate poly(dI-dC)·(dI-dC) or poly(dG-dC)·(dG-dC)). All reactions were carried out for 2 h at 37°C and quenched by heating for 10 min at 80°C. In separate series, substrate concentrations were varied in the range of 0.05–10 μM for cofactor. Samples underwent HPLC-MS/MS analysis as described below.

### MAT2A–DNMT3A cascade reactions with methionine analogue

The enzymatic cascade reactions were conducted in a 20 µl solution containing 10 μM MAT2A (I117A), 1 μM DNMT3A (WT, R887A or R887G), 6 μM poly(dI-dC)·(dI-dC), 25 mM Tris–HCl (pH 7.4), 5 mM MgCl_2_, 50 mM KCl, 1 mM ATP, utilizing a total amino acid (Met or N_3_-Met) concentration of 1 mM. Reactions were incubated for 4 h at 37°C and inactivated for 10 min at 80°C. Next, samples were centrifuged at 20 000 × *g* for 30 min at 4°C and 12 pmol of DNA were digested with P1 and subjected to HPLC-MS/MS analysis.

### Quantitative HPLC-MS/MS

50–3000 ng of DNA were enzymatically cleaved into nucleotides utilizing 0.01 U/μl of nuclease P1 (Sigma) in 40 μl of P1 reaction buffer (10 mM sodium acetate and 1 mM zinc acetate, pH 5.5) for 4 h at 50°C, succeeded by an overnight incubation with 0.01 U/μl of FastAP alkaline phosphatase (Thermo Fisher Scientific) at 37°C. Reactions were terminated by heating at 80°C for 10 min, then followed by centrifugation at 20 000 × *g* for 30 min at 4°C. Centrifuged samples were loaded into an integrated HPLC/ESI-MS/MS system (Agilent 1290 Infinity/6410 Triple Quad LC/MS) equipped with a Supelco Discovery HS C18 column (7.5 cm × 2.1 mm, 3 μm) using a linear gradient elution of solvents A (0.0075% formic acid in water) and B (0.0075% formic acid in acetonitrile) at a flow rate of 0.3 ml/min at 30°C as detailed below: 0–5 min, 0% B; 5–15 min, 10% B; 15–20 min, 100% B. The mass spectrometer functioned in positive ion MRM mode, recording the intensities of nucleoside-specific ion transitions: N_3_-m^5^dC 349.2→233.1, m^5^dC 242.1→126.1; dC 228.1→112.0, dG 268.1→152.1.

DNA cytosine modifications were quantified using standard curves established utilizing FastAP-treated nucleotides (Thermo Fisher Scientific) as standards: m^5^dC (linear range of 0.0125–0.8 pmol) and dC, dG, dT, and dA (0.38–12 pmol). Calibration of N_3_-m^5^dC was accomplished in the range of 0.2–25 fmol using a standard nucleoside obtained from enzymatically azidoalkylated 25-mer oligonucleotide hydrolysates, as described previously [29]. All signals were normalized to dG to account for variances in DNA input. MS data were analyzed with Agilent MassHunter software.

### Cell culture maintenance

Mouse embryonic stem E14TG2a cell line was purchased from the American Type Culture Collection of Authenticated Cell Cultures (ATCC CRL-1821). Cells were cultured on 0.15% gelatin-coated dishes in Dulbecco’s modified Eagle’s medium (DMEM; Gibco, USA) supplemented with 15% fetal bovine serum (Gibco, USA), 1× penicillin/streptomycin (Gibco, USA), 0.1 mM sodium pyruvate (Gibco, China), 0.1 mM β-mercaptoethanol (Gibco, USA), 1 mM L-alanyl-L-glutamine (Gibco, Japan), 1× non-essential amino acids (NEAA; Gibco, USA), 1000 U/ml mouse leukemia inhibitory factor (mLIF; Millipore, Germany), 3 µM CHIR99021 (Sigma–Aldrich, USA), and 1 µM PD0325901 (Sigma–Aldrich, USA) or in serum media without 2i.

### Generation of mouse embryonic *Dnmt3a* knock-in cells

Mouse embryonic stem E14TG2a cell lines harboring respective *Dnmt3a*^R887A^, *Dnmt3a*^R887G^, and *Mat2a*^I117A^ alleles were established using genome prime editing PE3 strategy as described previously [37]. pegRNAs and sgRNA were prepared as detailed below: 10 µM of complementary single-stranded pegRNA and sgRNA oligonucleotides (Metabion) were phosphorylated with 5 U T4 PNK (ThermoFisher Scientific) in PNK buffer containing 1 mM of ATP at 37°C for 30 min, then denatured at 95°C for 5 min, and annealed at a cooling rate of 0.1°C /s. pegRNA sequences were cloned into pU6-pegRNA-GG-acceptor (Addgene #132777) by incubating 2.5 nM of annealed oligonucleotides, 30 ng of vector, 5 U FastDigest Eco31I (ThermoFisher Scientific), and 5 U T4 DNA ligase (ThermoFisher Scientific) in ligase buffer at 37°C for 5 min, followed by incubation at 22°C for 5 min, repeating the reactions for 6 cycles. sgRNA oligonucleotides were cloned into phU6-gRNA (Addgene #53188) at BpiI sites.

Prior to transfection, a PE3 vector mixture was prepared in 25 μl of Opti-MEM medium (Gibco), including 750 ng of PE2-T2A-Puro, 250 ng of pegRNA, and 100 ng of sgRNA plasmids. 7.5 × 10^4^ E14TG2a cells were transfected using Lipofectamine LTX (Invitrogen) according to the manufacturer’s instructions. After 48 h, the cells were treated with 2 μg/ml puromycin for 2 days and then plated for clonal expansion into individual gelatin-coated wells in 96-well plates. After clone expansion, 1 × 10^5^ cells of each clone were collected and screened by direct PCR using a Phire Tissue Direct PCR Master Mix (ThermoFisher Scientific) and HhaI (for *Dnmt3a*^R887A^), PvuI (for *Dnmt3a*^R887G^), and SsiI (for *Mat2a*^I117A^) (ThermoFisher Scientific) restriction analysis, according to manufacturer’s guidelines to identify corresponding genome substitutions. Genome editing in the *Dnmt3a* and *Mat2a* loci was confirmed by Sanger sequencing. Primer sequences are given in Supplementary Table S1.

### Cell electroporation with Ado-6-azide

Cells were seeded in 24-well plates at a density of 5 × 10^4^ cells per well in LIF-supplemented DMEM and grown for 48 h. Each well was subsequently washed twice with Opti-MEM medium (ThermoFisher Scientific) and inoculated with 300 µl of a freshly prepared 1 mM Ado-6-azide solution in Opti-MEM medium. Electroporation was conducted using a NEPA21 electroporator (Nepa Gene) equipped with a CUY900 cell culture plate electrode, applying a cell poring pulse voltage of 200 V and a duration of 5 ms. After electroporation, cells were incubated for an additional 25 min at 37°C, replenished with fresh mESC medium, and incubated for an additional 3 h. Cell pellets from each sample were collected and preserved at −20°C.

### Metabolic labeling with methionine analogue

Cells were plated in 24-well plates at a density of 2.5 × 10^4^ cells per well in LIF-supplemented DMEM. After 48 h, cells were administered 1 mM of methionine analogue N_3_-Met in the DMEM media containing 200 µM methionine (Gibco), or in the methionine-depleted medium (Gibco) supplemented with 0–50 µM methionine (Sigma) and 200 µM L-cysteine (Sigma). Following 24 h of treatment, cells were harvested and stored at −20°C.

### gDNA isolation

Aproximately 10^6^ pelleted embryonic stem cells were resuspended in 200 μl of a 1:1 mixture of PBS and lysis buffer (0.2 M Tris–HCl, 0.25 M NaCl, 0.025 M EDTA, 0.5% SDS, pH 8; 1 mg/ml Proteinase K) and incubated for 40 min at 56°C. Samples were then subjected to RNase A (ThermoFisher Scientific) treatment for 10 min at room temperature, followed by genomic DNA isolation using a Genomic DNA Clean & Concentrator-10 Kit (Zymo Research) and kept at −20°C. Three micrograms of gDNA from each sample underwent HPLC-MS/MS analysis as described above.

### Preparation of Dnmt-TOP-seq libraries

Dnmt-TOP-seq libraries were prepared as described previously [29]. In brief, 900 ng of azide-tagged gDNA was sheared to 200 bp with a Covaris E220 sonicator. DNA fragments underwent end-repair using a DNA End Repair Kit (ThermoFisher Scientific) according to the manufacturer’s recommendations and were purified with GeneJet PCR Purification Kit (ThermoFisher Scientific). Subsequently, repaired DNA was 3′-A-tailed with Klenow exo- polymerase in Klenow buffer (ThermoFisher Scientific) in the presence of 0.5 mM dATP at 37°C for 45 min, then thermally inactivated at 75°C for 15 min and purified using GeneJet PCR Purification Kit. Partially complementary A1/A2 adapters (Metabion) were annealed and ligated overnight at 22°C with 15 U of T4 DNA Ligase (ThermoFisher Scientific) in ligase buffer, then inactivated at 65°C for 10 min, and purified with DNA Clean & Concentrator-5 column (Zymo Research). Next, DNA was combined with 20 μM biotinylated alkyne-containing DNA oligonucleotide (5′-T(alkyneT)TTTTGTGTGGTTTGGAGACTGACTACCAGATGTAACA-(biotin)-3′; Base-click) and 8 mM CuBr: 24 mM THPTA mixture (Sigma) in 50% of dimethylsulfoxide (DMSO), incubated for 20 min at 45°C, and purified with GeneJet NGS Cleanup kit (ThermoFisher Scientific). Biotinylated DNA was enriched by incubating with 0.1 mg Dynabeads MyOne C1 Streptavidin (ThermoFisher Scientific) in buffer A [10 mM Tris–HCl (pH 7.4) and 1 M NaCl] at room temperature for 3 h on a roller. After incubation, DNA-bound beads were washed twice with buffer B [10 mM Tris–HCl (pH 7.4), 3 M NaCl, and 0.05% Tween 20], twice with buffer A (supplemented with 0.05% Tween 20), and once with 100 mM NaCl. resuspended in water and heated for 5 min at 95°C to elute the enriched DNA fraction. The enriched DNA fraction was then eluted in water by heating at 95°C for 5 min. Recovered DNA was utilized in a priming reaction using 1 U Pfu DNA polymerase (ThermoFisher Scientific), 0.2 mM dNTP, and 0.5 μM complementary priming strand 5′-TGTTACATCTGGTAGTCAGTCTCCAAACCACACAA-3′ (with custom LNA modifications and phosphorothioate links at the 3′ end; Exiqon). The priming reaction was conducted under the following cycling conditions: 95°C for 2 min; followed by 5 cycles at 95°C for 1 min, 65°C for 10 min, and 72°C for 10 min. To amplify a primed DNA library, amplification reaction was prepared containing 20 µl of priming mixture, 50 µl of 2 × Platinum SuperFi PCR Master Mix (ThermoFisher Scientific), and barcoded fusion PCR primers A(Ad)-EP-barcode-primer (63 nt) and trP1(Ad)-A2-primer (45 nt) at 0.5 μM each (both primers contained phosphorothioate modifications). Thermocycler parameters were as follows: 94°C for 4 min; followed by 15 cycles at 95°C for 1 min, 60°C for 1 min, 72°C for 1 min. The MagJET NGS Cleanup and Size Selection Kit (ThermoFisher Scientific) was used to size-select final libraries for fragments of around 300 bp. The quality and quantity of libraries were assessed using an Agilent 2100 Bioanalyzer (Agilent). Libraries were subjected to Ion Proton (ThermoFisher Scientific) sequencing. Primer sequences are presented in Supplementary Table S2.

### Processing of Dnmt-TOP-seq data

mESC TOP-seq data were processed as described previously [29]. Quality of the data was evaluated using FastQC 0.11.9 (https://github.com/s-andrews/FastQC). Reads longer than 80 nt and containing the 5′ adapter sequence were retained followed by trimming of the 3′ adapter sequence using cutadapt 4.4 [38]. The sequence of the priming oligonucleotide and the genomic part at close proximity to the 5′ adaptor was then corrected using an in-house perl 5 script (http://www.perl.org/) to account for possible starting position shifting. Processed read sequences were mapped to the mouse genome using BWA-MEM tool [39] and only mappings with a quality score of ≥30 were retained using samtools (-q 30 -F 0 × 800 -F 256) [40] and clipped alignments were identified using samclip (https://github.com/tseemann/samclip) with default settings. Reads were then assigned to a nearest CG target and deduplicated (only one per group was retained) utilizing an in-house R 4.3.0 script (https://www.R-project.org) employing GenomicRanges [41], data.table (https://github.com/Rdatatable/ data.table), ShortRead [42], dplyr [43], Rsamtools [44], and gUtils (https://github.com/mskilab-org/gUtils) packages. Finally, CpG coverage was defined as a sum of all assigned reads on both strands and Dnmt-TOP-seq signal for each genomic element was calculated as a fraction of identified (coverage ≥ 1) CpG targets within the region of interest.

### Reference genome annotation

Mouse (*Mus musculus*) reference genome (GRCm38) sequence in fasta format was downloaded from GENCODE database [45]. Reference coding transcripts were retrieved from the NCBI database (https://ncbi.nlm.nih.gov/). RepeatMasker annotated repeats were obtained from the UCSC FTP. CGI, CGI shelves, and shores were retrieved using the annotatr R package [46]. Coding and non-coding gene promoters were downloaded from the EPD database [47]. Protein-coding genes and pseudogenes (processed and unprocessed) were extracted from the GENCODE genome annotation. Silencers were downloaded from silencersDB (http://health.tsinghua.edu.cn/SilencerDB). Enhancer were downloaded from EnhancersAtlas 2.0 [48], active and poised enhancer marks (ENCFF462XPD, ENCFF188LEW) and CTCF sites (ENCFF832AQF) were downloaded from the ENCODE project (https://www.encodeproject.org/). All other genomic elements were retrieved from the GENCODE genome annotation (M23).

### Functional enrichment analysis

Genomic element enrichment was computed by building a contingency table of all identified targets per each element and Fisher’s exact test was used to estimate the odds ratio and significance for the enrichment or depletion. Genomic features CpG modification profiles were created using EnrichedHeatmap [49] and plotted using ggplot2 [50]. 

Sequences flanking each selected CpG were extracted and used to generate sequence logos, with nucleotide frequencies represented as information content in bits. WEB logo plots were created using ggseqlogo package [51] using genome-wide CpG sites with coverage >4 and, where applicable, intersected with the genomic element of interest. The resulting logos were used to assess whether the CpGs analyzed showed any preferential sequence context.

### Statistical analysis

Quantitative HPLC-MS/MS data points denote the mean and standard deviation (SD) of three separate biological replicates. Differences between groups were assessed using *t* test, with statistical significance defined as *P* < 0.05 using GraphPad Prism 8.0. The statistical tests for bioinformatic analysis were conducted using R 4.3.0 as specified in the figure legends and method sections for each experiment.

### Data and code availability

All Dnmt3a-TOP-seq data generated in this study have been deposited in the NCBI Gene Expression Omnibus (GEO) under accession number GSE297788 and are publicly available at https://www.ncbi.nlm.nih.gov/geo/query/acc.cgi?acc=GSE297788. Publicly accessible mouse Dnmt1-TOP-seq datasets may be found in the GEO database with accession code GSE182445 and GSE267304. TAB-seq data were retrieved from GEO database (GSE36173). All code used for data processing, statistical analysis, and figure generation is available on Zenodo (DOI:10.5281/zenodo.18959359).

## Results

### Engineering DNMT3A for the catalytic transfer of extended moieties from synthetic AdoMet analogs

**Figure 1.**
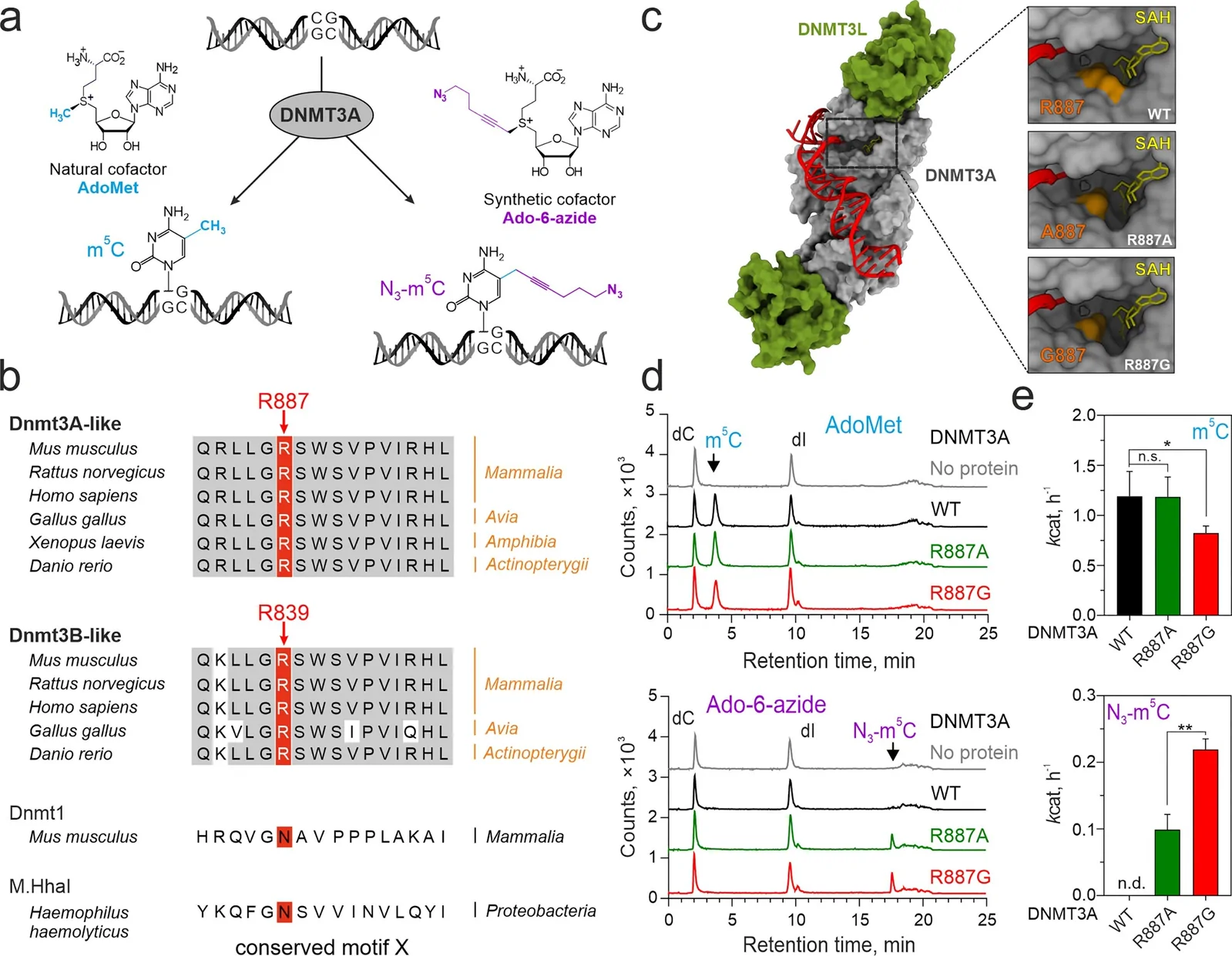
Steric engineering of DNMT3A methyltransferase enables catalytic activity with extended AdoMet analogues *in vitro*. (**A**) Enzyme-based modification of CpG sites by the mouse DNMT3A and DNMT3B *de novo* DNMTs. Biological methylation using the AdoMet cofactor produces 5-methylcytosine (m^5^C), whereas the bioorthogonal transfer of an extended functionalized side chain from a synthetic cofactor analog, Ado-6-azide, results in 5-(6-azidohex-2-ynyl)cytosine (N_3_-m^5^C) (depicted in cyan and magenta, respectively). (**B**) Conservation of the R887 and R839 residues in motif X across animal DNMT3A and DNMT3B-like proteins, as well as mouse DNMT1 or GCGC-specific bacterial M.HhaI methyltransferases. Identical amino acids are shown in gray boxes. (**C**) Structure of the catalytic/cofactor-binding pocket of DNMT3A WT (PDB: 6w8b) and engineered R887A and R887G variants. (**D**) Representative HPLC–MS/MS chromatograms of nucleosides derived from DNMT3A-modified poly(dI-dC)•poly(dI-dC) DNA. Reactions were conducted using 6 μM DNA substrate, 100 μM AdoMet or Ado-6-azide, and 800 nM DNMT3A variants for 4 h at 37°C. (**E**) Apparent turnover rates of DNMT3A variants with AdoMet and Ado-6-azide cofactors. Transferase activity was determined in triplicate reactions containing 6 μM poly(dI-dC)•poly(dI-dC) DNA substrate, 100 μM cofactor and 50 nM of each DNMT3A variant at 37°C for 2 h and analyzed as in Supplementary Fig. S2. Error bars denote ± SD. n.d. indicates not detected; n.s. not significant (*P* > 0.05); *P < 0.05; **P < 0.005.

Previously we reported that a single N1580A substitution in mouse DNMT1 catalytic domain efficiently conferred the mammalian enzyme with alkynyltransferase activity, enabling the preferred exploitation of synthetic AdoMet analogs, including the most widely applicable synthetic analog, Ado-6-azide (Fig. 1A), for DNA azidoalkylation *in vitro* and *in vivo* [29]. Given that DNMTs exhibit a highly conserved catalytic domain characterized by ten sequence motifs common to both eukaryotic and prokaryotic cytosine-5 methyltransferases, we consequently selected a respective arginine residue for alanine or glycine substitutions in motif X (Fig. 1B) to enlarge the cofactor pocket of DNMT3A (Fig. 1C). *In vitro* investigation of azidoalkylation activity included cloning and expressing the wild type and two engineered variants as his-tagged full-length proteins in the methylotrophic yeast *P. pastoris*, achieving high yield soluble forms of the corresponding enzymes (Supplementary Fig. S1A). For the assessment of their catalytic proficiency, we examined the obtained recombinant variants by comparing their activity with AdoMet cofactor or its synthetic analog, Ado-6-azide. We used polymeric DNA substrates poly(dI-dC)·poly(dI-dC) and poly(dG-dC)·poly(dG-dC), containing of repetitive CpI or CpG target sites, and a 25-mer oligonucleotide duplex, containing a single unmodified CpG site. HPLC-MS/MS analysis confirmed DNMT3A-selective catalysis using AdoMet and the extended analogue Ado-6-azide with all DNA substrates (Fig. 1D and Supplementary Fig. S1B). Kinetic characterization of the DNMT3A variants under steady-state conditions (Table 1; Fig. 1E and Supplementary Fig. S3) confirmed that in our hands the WT enzyme exhibited methylation activity (Fig. 1E and Supplementary Fig. S3A) similar to that described earlier [52]. In contrast to DNMT1 engineering [29], the alanine substitution minimally affected catalysis and affinity to AdoMet, whereas the R887G variant resulted in a reduced turnover rate and *K*_M_ values for the native cofactor. Conversely, the azidoalkylation activity of the DNMT3A WT with Ado-6-azide was below detectable threshold (Fig. 1E and Supplementary Fig. S3A). Notably, the R887G variant exhibited the highest azidoalkylation rate with Ado-6-azide on both DNA substrates, along with nearly an order-of-magnitude higher cofactor selectivity. Interestingly, both DNMT3A variants exhibited nearly identical affinity for the synthetic cofactor (apparent *K*_M_), suggesting that the improved catalytic proficiency might be attributed to a greater flexibility of the cofactor binding pocket rather than an enhanced binding.

**Table 1.** Apparent catalytic parameters of DNMT3A variants

DNMT3A	*k*_cat_^AdoMet^, h^−1^	*K*_M_^AdoMet^, μM	*k*_cat_/*K*_M_	*k*_cat_^Ado-6-azide^, h^−1^	*K*_M_^Ado-6-azide^, μM	*k*_cat_/*K*_M_	Selectivity to Ado-6-azide
poly(dI-dC)∙poly(dI-dC)							
WT	1.2 ± 0.25	0.36 ± 0.08	**3.3 × 10^6^**	**–**	**–**	**–**	
R887A	1.2 ± 0.20	0.30 ± 0.10	**3.9 × 10^6^**	0.10 ± 0.02	0.44 ± 0.12	**2.3 × 10^5^**	**0.06**
R887G	0.82 ± 0.07	0.72 ± 0.21	**1.1 × 10^6^**	0.22 ± 0.02	0.44 ± 0.10	**5.0 × 10^5^**	**0.45**
poly(dG-dC)∙poly(dG-dC)							
WT	0.29 ± 0.05	0.30 ± 0.07	**1.0 × 10^6^**	**–**	**–**	**–**	
R887A	0.27 ± 0.01	0.37 ± 0.12	**7.3 × 10^5^**	0.0076 ± 0.0020	0.32 ± 0.10	**2.4 × 10^4^**	**0.03**
R887G	0.10 ± 0.01	0.85 ± 0.29	**1.2 × 10^5^**	0.0186 ± 0.0003	0.42 ± 0.11	**4.4 × 10^4^**	**0.37**

**Figure 2.**
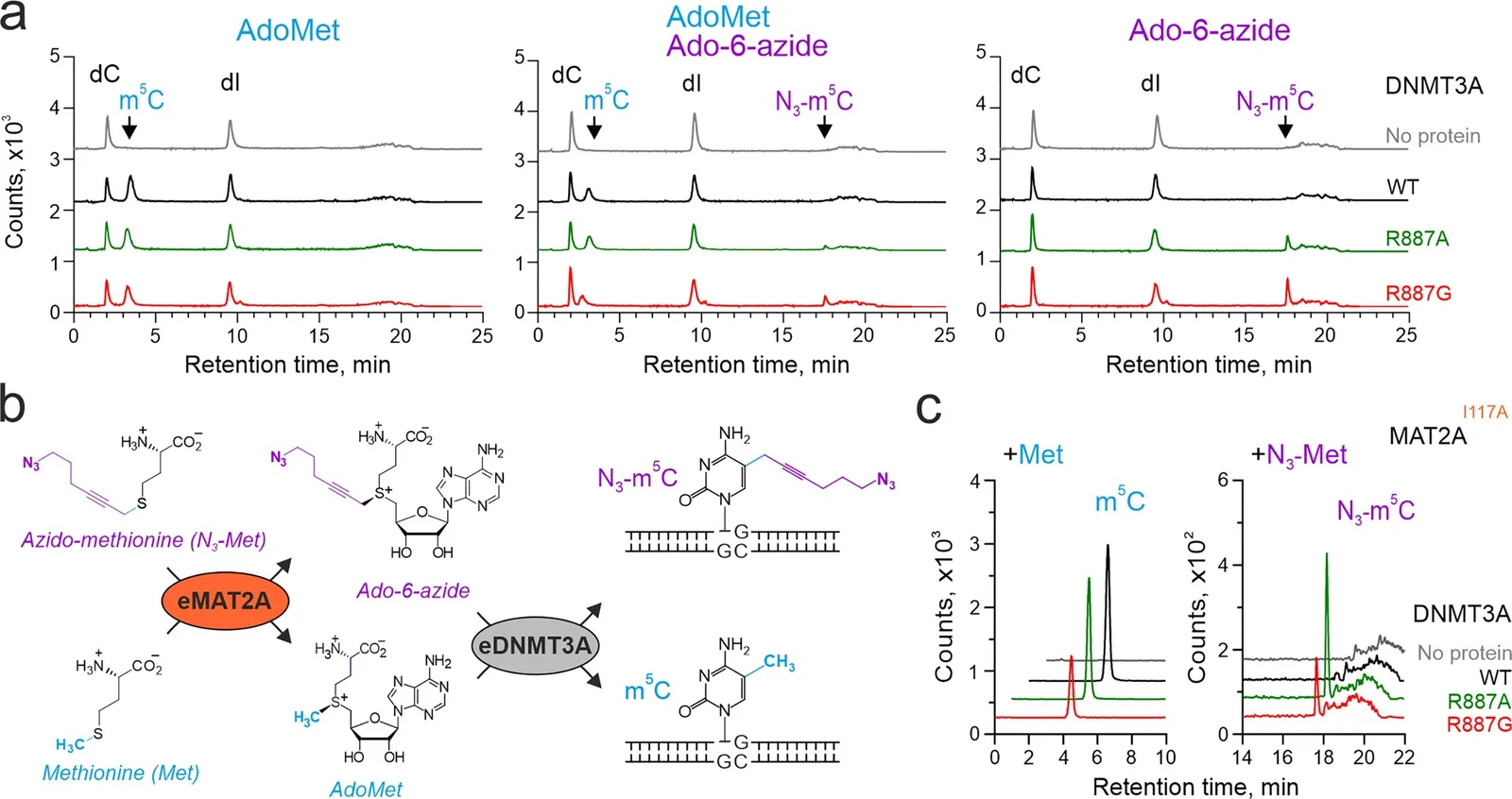
Cofactor selectivity of the engineered DNMT3A variants. (**A**) Comparative HPLC-MS/MS analysis of selective DNA modification by DNMT3A variants in the presence of AdoMet, Ado-6-azide, and their equimolar combination under steady-state conditions. DNA modification reactions were performed using 6 μM poly(dI-dC)·poly(dI-dC) DNA substrate, 800 nM DNMT3A variants, and 100 μM cofactor in total for 4 h at 37°C. (**B**) Experimental approach assessing DNA modifications in cascade reactions using engineered methionine adenosyltransferase (eMAT2A) and DNMT3A (eDNMT3A) variants in the presence of l-methionine (Met) or synthetic analogue azido-methionine (N_3_-Met). (**C**) Representative HPLC–MS/MS chromatograms illustrating DNA modification products resulting from the MAT2A^I117A^–DNMT3A cascade labeling. Cascade reactions were carried out using 6 μM poly(dI-dC)·poly(dI-dC) DNA substrate, 10 μM Mat2A, 1 μM DNMT3A variants, and 1 mM of L-methionine or azido-methionine for 4 h at 37°C.

A critical element for attaining the DNMT3A azidoalkylation activity in live cells is its ability to utilize the exogenous cofactor in the presence of endogenous AdoMet. Therefore, we next assessed the transfer of the azide tags onto DNA at equimolar amounts of both cofactors present in the reaction. HPLC-MS/MS analysis found that the extended cofactor was preferentially used by both engineered variants (Fig. 2A and Supplementary Fig. S2C), whereas the R887G variant gave a higher yield of the azidoalkylation products. This clearly illustrated the potential of the DNMT3A variants to render deposition of bioorthogonal azide groups onto DNA in the presence of endogenous AdoMet inside mammalian cells.

Considering that designer MAT2A proteins can facilitate efficient biosynthesis of cofactor analogues both *in vitro* and *in vivo* [31, 33], we subsequently examined the performance of the DNMT3A variants in cascade reactions with an engineered variant of MAT2A (I117A) (Fig. 2B), which has previously been used for DNMT1-selective metabolic genome labeling with a corresponding methionine analog, N_3_-Met [33]. Our cascade experiments revealed that the DNA methylation activity of the DNMT3A variants (Fig. 2C) was in good agreement with their kinetic parameters observed with AdoMet (Fig. 1D). As anticipated, cascades with both engineered variants demonstrated a clearly measurable transfer of the extended azide moiety from N_3_-Met onto DNA (Fig. 2C). Altogether, these findings clearly suggested that the engineered DNMT3A variants could also be active with the metabolically produced Ado-6-azide cofactor in living cells.

### Engineering ES cells for DNMT3A-mediated genome tagging *in cellulo*

**Figure 3.**
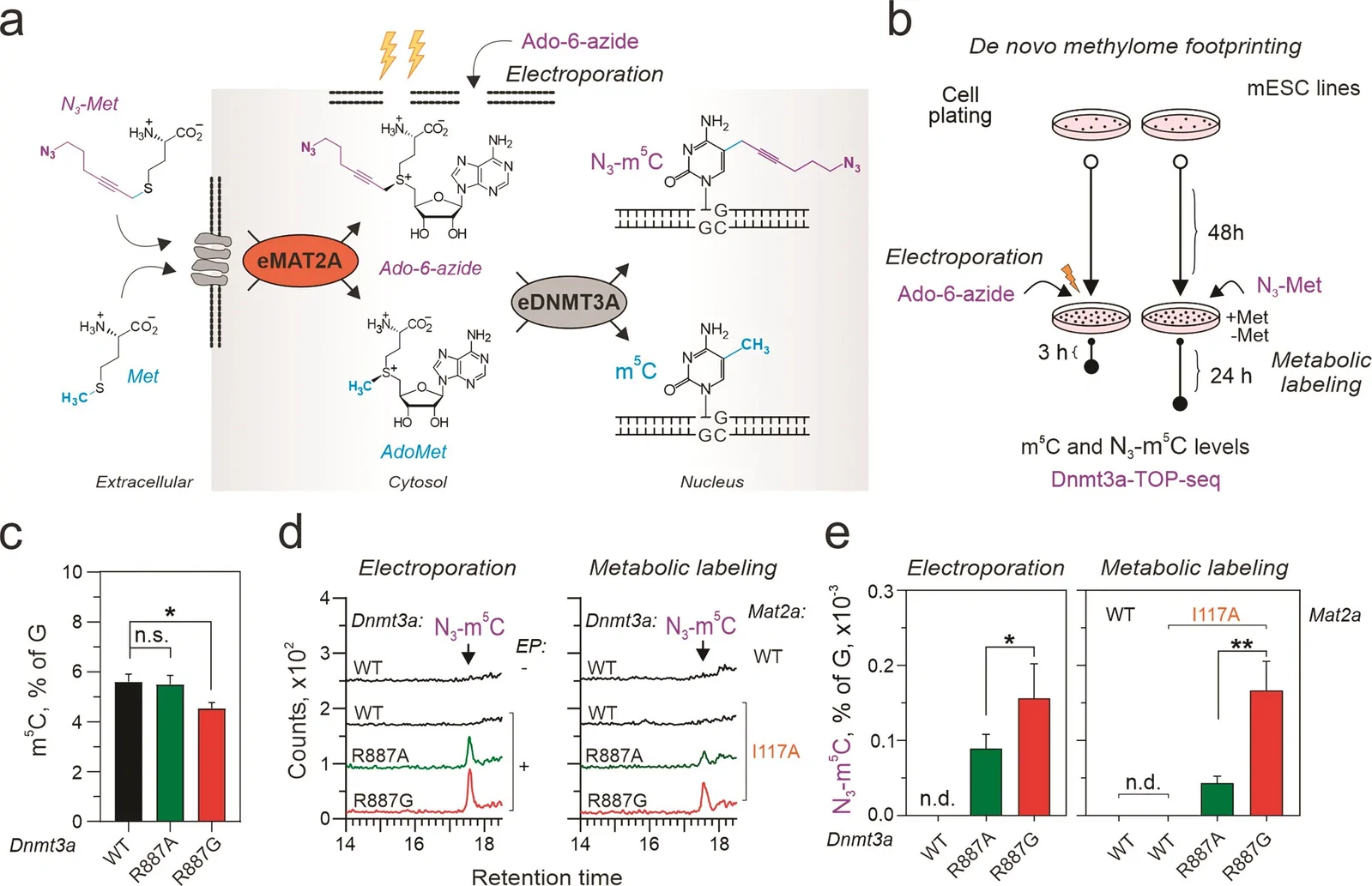
DNMT3A-dependent chemical tagging of genomic DNA in live mESCs. (**A**) Strategy for DNMT3A catalysis-dependent bioorthogonal genome labeling in live mESCs using either pulse-electroporation with the synthetic cofactor Ado-6-azide or the propagation of cells with the cell membrane-permeable synthetic methionine analog Azido-methionine (N_3_-Met). (**B**) Experimental workflow to characterize genome-wide DNMT3A-specific cytosine modifications in engineered mESCs. Genome pulse labeling was carried out for 3 h after electroporation with 1 mM Ado-6-azide. Metabolic genome labeling was conducted by exposing cells to 1 mM N_3_-Met for 24 h under either normal (+Met) or methionine-depleted (-Met) conditions. (**C**) HPLC-MS/MS analysis of global m^5^C levels in engineered mESCs assessed as % of dG. Bars represent mean ± SD, *n* = 3 independent experiments. (**D**) HPLC-MS/MS analysis of genomic cytosine modification in *Dnmt3a*^WT^, *Dnmt3a*^R887A^, and *Dnmt3a*^R887G^ mESCs after pulse labeling with 1 mM of Ado-6-azide or metabolic genome labeling with 1 mM N_3_-Met. (**E**) Levels of DNA azide-tagging in the engineered mESC lines following genome labeling. Bars denote the mean expressed as % of dG ± SD, *n* = 3 independent experiments; n.d. – not detected.

To implement a DNMT3A-selective bioorthogonal genome tagging system in mammalian cells (Fig. 3A), we first integrated the corresponding codons (R887A or R887G) in exon 23 of the *Dnmt3a* gene. Taking into account that DNMT3A in general is substantially less proficient than DNMT1 and that the engineered variants displayed even lower catalytic rates with the synthetic cofactor *in vitro* (Table 1), we decided that efficient genome tagging would be best achieved in cells with knock-in substitutions on both alleles. Cas9 nickase-RT-based search-and-replace editing [37] (Supplementary Fig. S4A) yielded biallelic edits with an efficiency of 10% and 24% for R887A and R887G, respectively (Supplementary Fig. S4C). These cell lines were further analyzed and proved instrumental for DNMT3A catalytic tracking using electroporation (see below). To complete the installation of full metabolic labeling cascades in mESCs, three randomly selected clones from each of the DNMT3A biallelic KI cell line were subjected to a second round of prime editing at the *Mat2a* gene to replace the I117 coding sequence with an alanine codon (Supplementary Fig. S4B). Monoallelic *Mat2a* substitutions were generated with an approximately 10% efficiency and were further verified by sequencing of corresponding loci to contain both intended substitutions.

The behavior of both types of the engineered mESC lines was explored in the context of physiological levels (0.2 mM) of Met in LIF-enriched medium after electroporation-based pulse-internalization of the synthetic cofactor Ado-6-azide or after exposure to the extended amino acid analogue N_3_-Met, respectively (Fig. 3B). HPLC-MS/MS analysis revealed nearly identical amounts of genomic m^5^C across *Dnmt3a*^WT^ and homozygous *Dnmt3a*^R887A^ KI cells, whereas the homozygous *Dnmt3a*^R887G^ cells exhibited 20% lower m^5^C levels (Fig. 3C), mirroring the DNMT3A catalysis pattern observed *in vitro* (Fig. 1E). Concurrently, the monoallelic I117A knock-in in *Mat2a* showed nearly no change in the m^5^C levels (Supplementary Fig. S5A), aligning well with prior findings in the engineered cellular MAT2A–DNMT1 cascade [33]. Remarkably, we found a well detectable accumulation of genomic N_3_-m^5^C in both engineered cell lines upon administration of the synthetic cofactor or its precursor (Fig. 3D and E). Control *Dnmt*^WT^ cell lines possessing the *Mat2a*^WT^ or *Mat2a*^WT/I117A^ alleles showed no detectable N_3_-m^5^C, demonstrating that both engineered genes were necessary for the metabolic genome tagging *in vivo*. In line with the observed catalytic proficiency of the DNMT3A mutants *in vitro* (Fig. 1E), *Dnmt3a*^R887G^ mESCs exhibited a 2–4-fold higher azidoalkylation activity as compared to the R887A cells in both labeling regimens.

Methionine restriction is known to negatively affect the cellular methylation potential and has been associated with altered aging [53, 54], disease progression and therapeutic efficacy [55, 56]. We therefore looked at the impact of methionine availability on the DNMT3A-specific genome labeling in the engineered ES cells. Indeed, we found that reduction of methionine concentration in the culture medium leads to global genome hypomethylation but increased labeling efficiency in both engineered cell lines (Supplementary Fig. S5), in line with diminished competition from the native substrate in the cellular cofactor/precursor pool. Importantly, while the parental (WT) mESCs exhibited no detectable genome azidoalkylation under any conditions tested, we observed minor DNA tagging in the *Mat2a*^WT/I117A^ control cells cultivated in the absence of Met. Taking into account some promiscuous WT DNMT1 alkylation activity *in vitro* [29], one should be aware of potential DNMT1-mediated DNA tagging in cells cultivated under conditions of severe Met limitation, which may interference with an accurate determination of the DNMT3A-dependent activity.

### Genome-wide chemical footprint of DNMT3A catalysis in live ES cells

**Figure 4.**
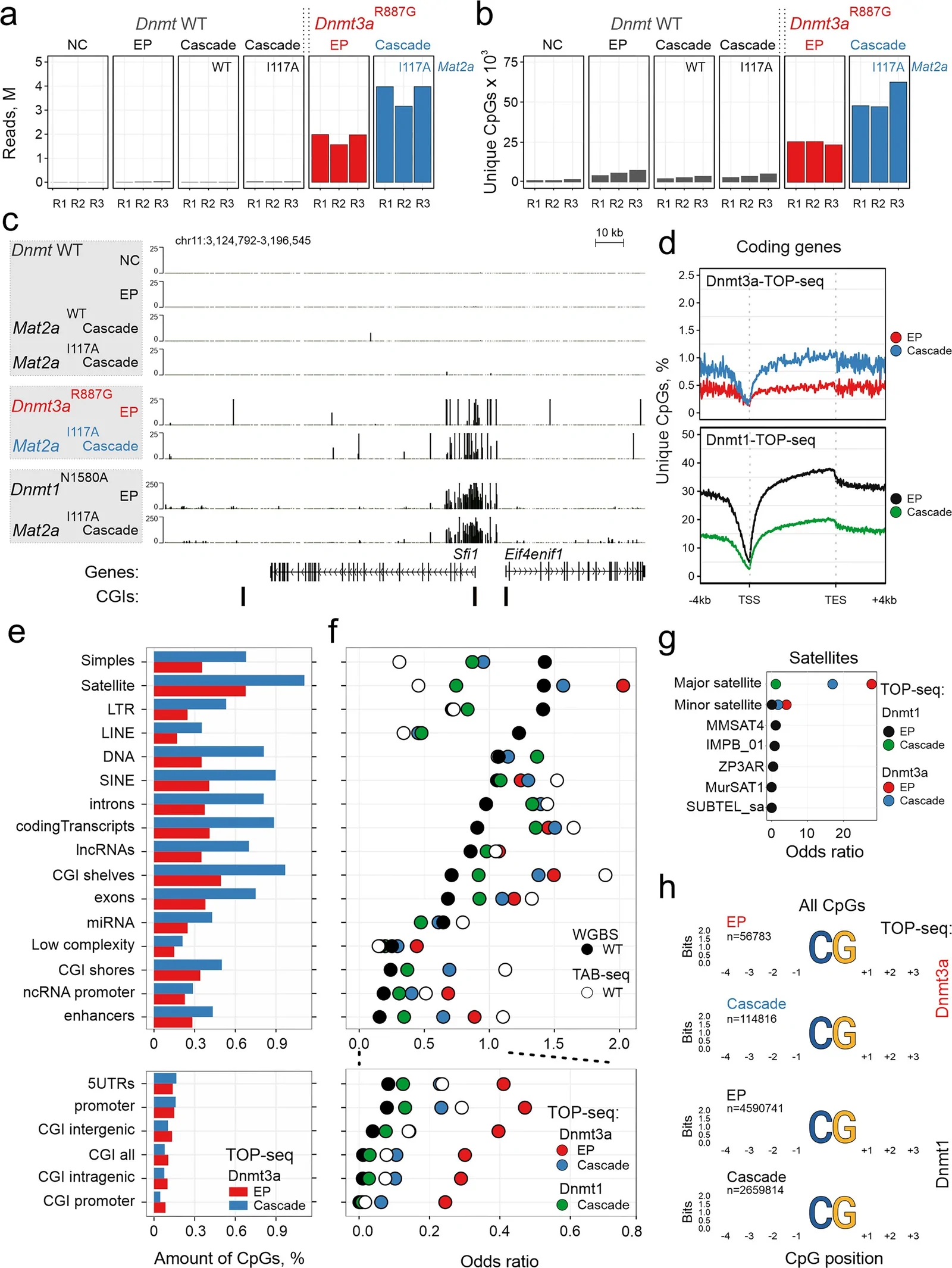
Analysis of DNMT3A-specific genome modification profile in mESCs using Dnmt-TOP-seq. (**A**) Total count of mapped reads (read start within 2 nt of CpG) identified in the Dnmt-TOP-seq libraries derived from *Dnmt* WT, *Dnmt3a*^R887G^, and *Dnmt1*^N1580A^ mESCs, 3 h post-electroporation with 1 mM Ado-6-azide (EP) or 24 h metabolic genome labeling with 1 mM azido-methionine (Cascade). Cascade labeling was performed in *Mat2a*^WT^ mESCs or cells with a single *Mat2a*^I117A^ allele. (**B**) A total number and percentage of unique CpGs in Dnmt3a-TOP-seq replicates. (**C**) Representative genome browser visualization of DNA modification patterns in mESC lines derived from Dnmt-TOP-seq libraries. Each track represents modification coverage values of individual CpGs. Genes and CpG islands (CGI) are shown below the tracks. (**D**) Distribution of CpG modification along coding genes, including 4 kb flanking regions. (**E**) Comparative distribution of unique CpGs modified by DNMT3A across genomic elements and regulatory features. (**F**) Comparative enrichment analysis of genomic attributes. The odds ratio indicates whether a particular genomic element is enriched (>1) or reduced (<1) in the Dnmt-TOP-seq, WGBS (*Dnmt* WT), and TAB-seq (*Dnmt* WT) datasets. (**G**) Enrichment analysis of satellite regions in the Dnmt-TOP-seq datasets. (**H**) Analysis of the flanking sequence preferences of DNMT3A and DNMT1 catalysis in genome-wide context in live mESCs. n denotes a number of unique CpGs in Dnmt-TOP-seq libraries.

The R887G variant showed a clearly stronger DNA labeling than R887A both upon pulse-electroporation with Ado-6-azide and when exposed to the Met analogue (Fig. 3D and E) and therefore the corresponding cell lines were chosen for further genomic studies of DNMT3A catalysis *in vivo*. Genomic maps of DNMT-specific catalytic events were determined using the TOP-seq (Tethered Oligonucleotide-Primed sequencing) technique, which exploits the deposited genomic azide tags as “click” handles for attaching an oligonucleotide to enable non-homologous priming of sequencing amplicons at the tagged sites [28]. TOP-seq reads (80–300 nt) containing a terminal CpG site and its 5′-adjoining region permit their unequivocal genomic mapping and thus identify the number and locations of the azide-tagged CpGs in the genome [28, 29, 33]. We assembled triplicate Dnmt3a-TOP-seq libraries from the LIF-cultivated engineered *Dnmt3a*^R887G^ or *Mat2a*^WT/I117A^  *Dnmt3a*^R887G^ mESCs (∼1 M cells per replicate) subjected to the respective labeling regimen (Fig. 3B and Supplementary Fig. S6) along with similarly treated *WT* and monoallelic *Mat2a*^I117A^ lines serving as controls. Here again, we found that the number of sequencing reads obtained from the azide-tagged engineered cells (98–126 M per biological replicate) was 2 orders of magnitude higher than that in the controls (Supplementary Tables S3 and S4). The on-target mapping (2–4 M mapped reads per replicate) and the number of unique identified CpGs in the metabolically treated *Mat2a*^WT/I117A^  *Dnmt3a*^R887G^ mESCs (Fig. 4A and B) was 2-fold higher than in the pulse-tagged *Dnmt3a*^R887G^ cells. The mean coverage of identified CpGs was around 70 × per replicate representing 0.3% of mapped genomic CpGs (Supplementary Fig. S8A and B). On the other hand, substantial amounts of detected CpGs in the electroporated WT control suggested that the DNMT3A-specific azidoalkylation profiles may be affected by the activity of WT DNMTs, particularly at peaking intracellular concentrations of Ado-6-azide following electroporation. To illustrate the specificity of the genome tagging, we looked at the distribution of TOP-seq peaks across a genomic region encompassing the spermatogenesis-related *Sfi1* gene. In this case, both engineered *Dnmt3a* cell lines showed a characteristic modification profile at CGIs associated with the *Sfi1* promoter, along with the respective DNMT1 maintenance activity (Fig. 4C), standing clearly above randomly scattered sparse peaks in the control lanes. Statistical analysis of biological replicates showed good concordance of the DNMT3A-TOP-seq data across protein-coding regions but limited convergence on the whole-genome scale (Supplementary Figs S6 and S7) suggesting that faithful discrimination at individual sites is only partially achievable at this coverage level. The DNMT activity at non-CpG sites could not be well discerned using the TOP-seq mapping at this tagging intensity, and will not be discussed further.

We next evaluated the genome-wide distribution of identified CpG sites in the Dnmt3a-TOP-seq libraries and compared them with Dnmt1-TOP-seq modification patterns previously determined in DNMT1-engineered cells [29, 35]. A clearly notable distinction was a much higher count of mapped reads and identified CpGs observed in the DNMT1- versus DNMT3A-engineered cells, in line with their markedly different global N_3_-m^5^C levels (Fig. 4D). Analysis of the CpG modifications along coding genes illustrated a quite similar distribution in modified CpGs in the engineered *Dnmt3a*^R887G^ cells across a variety of genomic features (Fig. 4D and Supplementary Figs S7A,B and S8C). Notably, the metabolic labeling produced almost double amounts of modified CpGs as compared to the pulse-tagged cells across all genomic elements, except for CGIs (Fig. 4E). The latter elements are strongly undermethylated and thus low DNMT3A modification levels and/or promiscuous activity of the WT DNMTs at peak cofactor concentrations may lead to relatively large errors in signal estimates. Another intriguing aspect was the inverted response to the labeling regiment (metabolic versus EP) by the DNMT1- versus DNMT3A-specific cells. Apart from biological interactions and cell-cycle–dependent expression profiles, enzymatic properties of the corresponding DNMTs, namely, a substantially higher intrinsic catalytic rate of DNMT1 may translate into an enhanced DNA tagging during a relatively brief but profound surge of the cofactor concentration following EP internalization.

### Functional disentanglement of individual DNMT contributions

To derive the catalytic contributions of individual DNMTs, we compared the determined TOP-seq data with a series of other relevant datasets using functional enrichment analysis, which helped to minimize the influence of mismatches of data formats and thus rectify signals reflecting genuine processes. We first looked at the two DNMT3A-specific TOP-seq profiles (pulse and metabolic) and the previously derived DNMT1-TOP-seq (metabolic) profiles (Fig. 4F and Supplementary Fig. S7C). In the absence of DNMT3B-selective data, we also included whole-genome bisulfite sequencing (WGBS) profiles of WT ESCs, which represented an aggregate m^5^C signal shaped by the activity of all three DNMTs and the three TET dioxygenases. Inspection of the comparative plot indicated that the DNMT3A activity was enriched in CGIs and its shores and shelves, as well as in promoter-proximal genomic regions, which was markedly higher than in the DNMT1-tagged cells and WGBS (Fig. 4F). Taking into account that the deposited azide tags are poor substrates for the TET enzymes [57], an enhanced enrichment of the DNMT-TOP-seq signal over WGBS may mark regions that undergo rapid methylation/TET-dependent demethylation turnovers leading to low steady-state levels of m^5^C. To verify this assumption, we added a published TAB-seq profile representing hm^5^C-enriched regions [58], which lent further support for active demethylation occurring in CGIs shores and shelves, coding transcripts, introns, exons, promoters, and enhancers. CGIs also showed a similar trend, but, given their low methylation levels, low enrichment values and possible cross-reactivity from WT methylation writers, a similar prediction would be less reliable.

Strikingly, the most prominent enrichment in DNMT3A targets was observed in satellite sequences and low complexity repeat sequences (Fig. 4F), which also showed a strong overall methylation (WGBS), but rather weak Dnmt1 and TAB-seq (hm^5^C) enrichment. This can well be interpreted as elements of profound DNMT3A activity. A closer look at individual satellite families identified major satellites as the major targets of the DNMT3A activity (Fig. 4G), highlighting a distinctive role of DNMT3A in the establishment of DNA methylation at these repeat sequences.

We also identified genetic features, such as LTR, simple repeats, and LINEs, that showed weak DNMT1, DNMT3A, and TAB-seq enrichment but strong global methylation (enriched WGBS). In the context of seemingly low DNMT1, DNMT3A, and TET activities, DNMT3B appeared to be the most probable contributor to methylation of these genomic elements. Since no direct data are yet available, it cannot be excluded that DNMT3B plays certain roles in methylation of other elements as well along with DNMT3A and DNMT1, which needs to be confirmed by further studies.

We also looked at the flanking context of genomic targets in the Dnmt3a-TOP-seq datasets in light of reported discrimination of CpG flanking sequences by DNMT3A and DNMT3B [34]. Both enzymes were reported to exhibit a strong but distinct preference for AT-rich flanks over GC-rich ones and show 100-fold differences in the methylation rates between preferred and disfavored sequences *in vitro* and in model cell systems. Contrary to these reports, with both types of labeling, the DNMT3A engineered cells showed no discernable nucleotide preferences within a −4 to +3 flanking window genome-wide or in specific genomic elements, including coding genes, CGIs or LTR repeat sequences (Fig. 4H and Supplementary Fig. S8F). This fully aligns with our observed lack of sequence bias for the DNMT1 activity *in vivo* (Fig. 4H and Supplementary Fig. S8F), suggesting that intrinsic preferences of the DNMT enzymes might be largely masked by interactions with internal factors within the native cellular milieu.

## Discussion

By integrating principles of chemical genetics, the development of bioorthogonal bump-and-hole toolkit enables catalysis-based interrogation of individual epigenetic writers in living systems—a capability that has remained largely inaccessible due to a lack of minimally perturbing approaches for manipulating DNMT activity within native cellular context. Prototype systems were previously proposed for AdoMet analog-based profiling of protein methylation targets in cell lysates [59], as well as analysis of histone [31] and mRNA adenine-*N*6 methylation [60, 61] in transfected and/or methionine-deprived cells. Recent advances in genome editing, notably CRISPR/Cas-based prime editors [37], have permitted direct installation of “hole” mutations at endogenous MTase loci, preserving physiological expression, localization, and regulatory control of the engineered enzyme while conferring in-cell biorthogonal reactivity. Leveraging this capability, in this study we present a genetically stable cellular system for selective catalytic tracking of a *de novo* DNMT in live cells, extending the chemical-genetic interrogation of DNA methylation writers under near physiological conditions.

The DNMTs share a highly conserved catalytic domain comprising ten sequence motifs found across eukaryotic and prokaryotic DNA C5-MTases. Notably, although similar repurposing of bacterial orthologs generally required dual substitutions in motifs IV and X [62, 63], a single N1580A mutation in motif X was sufficient to reverse the cofactor preference in DNMT1 [29]. Guided by sequence alignment and structural information, we identified residue R887 in DNMT3A as a key position whose substitution to a glycine yielded robust azidoalkylation activity and selectivity toward Ado-6-azide (Figs 1 and 2). These observations illustrate a remarkable plasticity of the active site of C5-MTases with regards to artificial and evolutionary (i.e. carboxy-SAM [64]) manipulation of the size of the catalytically transferable moieties.

On the other hand, a 2–3-fold reduction of the methylation rate compared to WT (Table 1) caused by the R887G mutation leads to a 20% drop in the genomic m^5^C level of the engineered mESC line as compared to the WT cells (Fig. 3C). WGBS studies of the DNMT3A KO cells showed a 40% drop in total CpG methylation [26] suggesting that the DNMT3A methylation is somewhat attenuated in our engineered mESCs representing a midway between the WT and the KO cells. Comparative enrichment analysis of the WT and DNMT3A KO methylation (Supplementary Fig. S9) indicates their close similarity across most of the genomic elements, except in highly methylated elements such as LTR, satellites and simples (see discussion of the latter below). To this end, we have previously observed the DNMT1-TOP-seq genomic profiles to be quite similar in lines containing mono- or bi-allelic DNMT1 mutations despite of even greater changes of the DNA methylation levels [35]. Therefore, one can reasonably assume that the slightly attenuated DNMT3A methylation profile in the mutant ESC line will lend itself for DNMT3A-directed azidoalkylation of natural target sites in the presence of the AdoMet analog, serving as the closest approximation of a native biological system to date.

Indeed, we show that bioorthogonal DNMT3A-mediated genome labeling could be achieved via pulse-internalization of exogenous Ado-6-azide or its metabolic production from a membrane-permeable precursor azido-methionine in CRISPR-engineered mESCs under nearly native conditions (Fig. 3). Each delivery approach offers unique benefits and limitations. Direct supplementation with Ado-6-azide enables robust temporal control of intracellular DNMT3A activity under saturating cofactor conditions. However, the labeling timing is confined to windows of 2–6 h, since the azide cofactor is rather unstable under physiological conditions. In contrast, feeding cells with an azido-methionine precursor relies on native transport and metabolic conversion to achieve a steady intracellular supply of Ado-6-azide. This allows prolonged DNMT-selective genome labeling under physiological conditions, albeit at the expense of temporal resolution. The presented TOP-seq data demonstrate that the metabolic approach affords a 2-fold higher accumulation of modified CpGs than the pulse delivery of the synthetic cofactor (Fig. 4B, D, and E). The attained tagging density of 0.0003%, in the context of nearly 6% methylation (Fig. 3C and E), means that on average only 1 in 20 000 m^5^C residues (or 1 in 0.5 M nucleotides) contains a bioorthogonal chemical extension, which is thus unlikely to cause detectable interference with the native cellular processes during the lifetime of the measurements.

Using comparative enrichment analysis, selective traces of the DNMT catalysis can be harnessed for facile discovery of genomic regions undergoing elevated or attenuated methylation/demethylation activity. In the context of relatively low m^5^C enrichment, the enhanced Dnmt3a-TOP-seq signal is expected to mark genomic regions undergoing competing methylation and TET-dependent demethylation. To this end, we found pronounced enrichment of the DNMT3A activity in genomic loci associated with CGIs, enhancers and promoter-proximal regions in pulse-tagged cells, which were also enriched in hm^5^C (Fig. 4F). The identified regions coincide with developmentally poised promoters and enhancers, where DNMT3A has been shown to act in opposition to TET1 and TET2, which maintain a hypomethylated state [65–67]. Moreover, TET1 may also have additional functions to restrict DNMT3A-mediated methylation spreading from methylated edges into CGIs, which was previously observed for differentiated cells [68]. Similar enrichment trends were also observed within CGIs themselves (Fig. 4F, lower panel), but their inherently low methylation levels may suggest that the active equilibrium is a background process and that other mechanisms (such as PRC binding) are largely responsible for maintaining the undermethylated state. Altogether, our data position DNMT3A, along with DNMT1 [35], as a pivotal player in the maintenance of regulatory genome elements at steady-state in mESCs, countered by active demethylation, and demonstrate how DNMT-selective tracking can identify and map dynamic DNA modification turnover processes to specific genomic loci.

Here, we also find a striking enrichment of the DNMT3A activity at major satellite sequences (Fig. 4G) in the context of relatively high m^5^C and low DNMT1 and hm^5^C enrichment, pointing to specific targeting within pericentric heterochromatin. Given the repetitive nature of centromeres, CpG sites within centromeric DNA exhibit a significant enrichment in methylation, contributing to the epigenetic establishment of centromere identity and stability [69, 70]. However, the contribution of individual DNMTs remains debated. Genetic evidence indicates that *Dnmt3b* deficiency primarily affects minor satellites, whereas *Dnmt3a* loss has limited impact on these regions [71], consistent with our observation, which indicated a barely detectable enrichment of DNMT3A modifications in the minor regions. The distinct specificity of *de novo* methyltransferases for major or minor satellites is further supported by studies of the distribution of preferred methylation targets [34] and the preferential association of DNMT3A rather than DNMT3B to pericentromeric regions [72]. Altogether, our findings corroborate the existence of this phenomenon and illustrate the capability of our chemical tagging approach to provide important insights into DNMT3A-mediated centromere functionality. Finally, comparatively low DNMT1, DNMT3A, and hm^5^C enrichment at LTRs, simples and LINEs (Fig. 4F) may point to an important role of DNMT3B methylation at these repeat sequences, in line with a pronounced reduction in DNA methylation observed at LINEs in *Dnmt3b* KO mouse embryonic fibroblasts [73]. Recent DNMT KO studies point at collective involvement of all three DNMTs in methylation of DNA, LINE, LTR, and simple repeat regions [74].

Several studies reported that the DNMT and the TET enzymes modify target sites situated within distinct sequence contexts with varying efficiencies *in vitro* and also exert pronounced preferences for some flanking contexts over others *in cellulo* [34, 75, 76]. Unexpectedly, our analyses of the individual DNMT3A or DNMT1 catalysomes (all identified target sites with coverage of ≥4) found no apparent preference with regards to CpG flanking sequences in primed ES cells (Fig. 4H). Structural analyses of the DNMT3A–DNA complexes at the atomic level [34] identified no direct contacts or other interactions of the R887 side chain with bound DNA that would implicate its involvement in defining sequence-specific preferences during catalysis. Thus one would not expect that the R887G point mutation could have a strong effect on the sequence specificity of the enzyme. The same consideration holds true for the DNMT1 N1580A variant as well [75]. A characteristic feature of the DNMT-TOP-seq approach is that only a small fraction of the target sites is labeled during analysis, and thus this “tip of the iceberg” view of the methylome may not fully reflect all subtle aspects of the catalytic behavior *in vivo*. Notably, this subtle bioorthogonal signal, being largely resistant to TET oxidation, is by definition different from the aggregate DNA methylation, which is shaped by the action of the three DNMT and three TET enzymes. To extract a specific enzymatic signature, gene knockouts or overexpression of individual DNMTs are often required [34, 75, 76] leading to extensive perturbations of the methylome [12, 17] and thus altered cellular environment and potentially disturbed behavior of the DNMT in question. Another point is that for the intrinsic catalytic selectivity to persist in the genomic profile, the rate-limiting step for catalysis must be the same as on naked DNA; however, this will not hold true in many diverse biological circumstances. The intracellular catalysis of DNMT3A and the other DNMTs is controlled by reversible protein modifications, interactions with chromatin and numerous regulatory factors [4, 22, 77], underscoring their integration into complex epigenetic networks. Hence, the lack of a detectable context sensitivity (Fig. 4H), besides some peculiarities of the data sampling discussed above, may reflect an extensive chromatin-level regulation that dominates the DNMT recruitment, effectively masking intrinsic DNA sequence preferences of the enzyme. Consequently, one may consider looking at the flanking sequence selectivity *in vivo* as a cell state- or cell type-dependent characteristic rather than a universal feature firmly casting genomic modification landscapes.

In conclusion, our findings underscore the potential of integrating orthogonal cofactor chemistry with intracellular metabolism to enable precise, real-time, and context-sensitive probing of DNMT3A-specific DNA methylation in live cell models under near-physiological conditions. Collectively with the complementary DNMT1-engineered cell lines, we present a versatile platform for catalytic untangling of individual methylation writers at the cellular, tissue and organismal levels.

## Supplementary Material

gkag906_Supplemental_File

## Data Availability

All the Dnmt3a-TOP-seq data underlying this article are available in the NCBI Gene Expression Omnibus (GEO) and can be accessed with accession number GSE297788. The mouse Dnmt1-TOP-seq datasets used in this work can be retrieved at GEO under accession numbers GSE182445 and GSE267304. The TABseq data used in this work were retrieved from GEO (GSE36173). All code used for data processing, statistical analysis, and figure generation is available on Zenodo (DOI:10.5281/zenodo.18959359).
