# Supplementary material for: Live-cell DNMT3A “catalysome” mapping using engineered methyl-transfer pathways
Source: Nucleic Acids Res. 2026 Sep 22;54(18):gkag906. doi: 10.1093/nar/gkag906 (PMC13594970; doi:10.1093/nar/gkag906)
Supplement: Supplementary file 1 [file gkag906_supplemental_file.pdf]

Supplementary Figures S1-S9

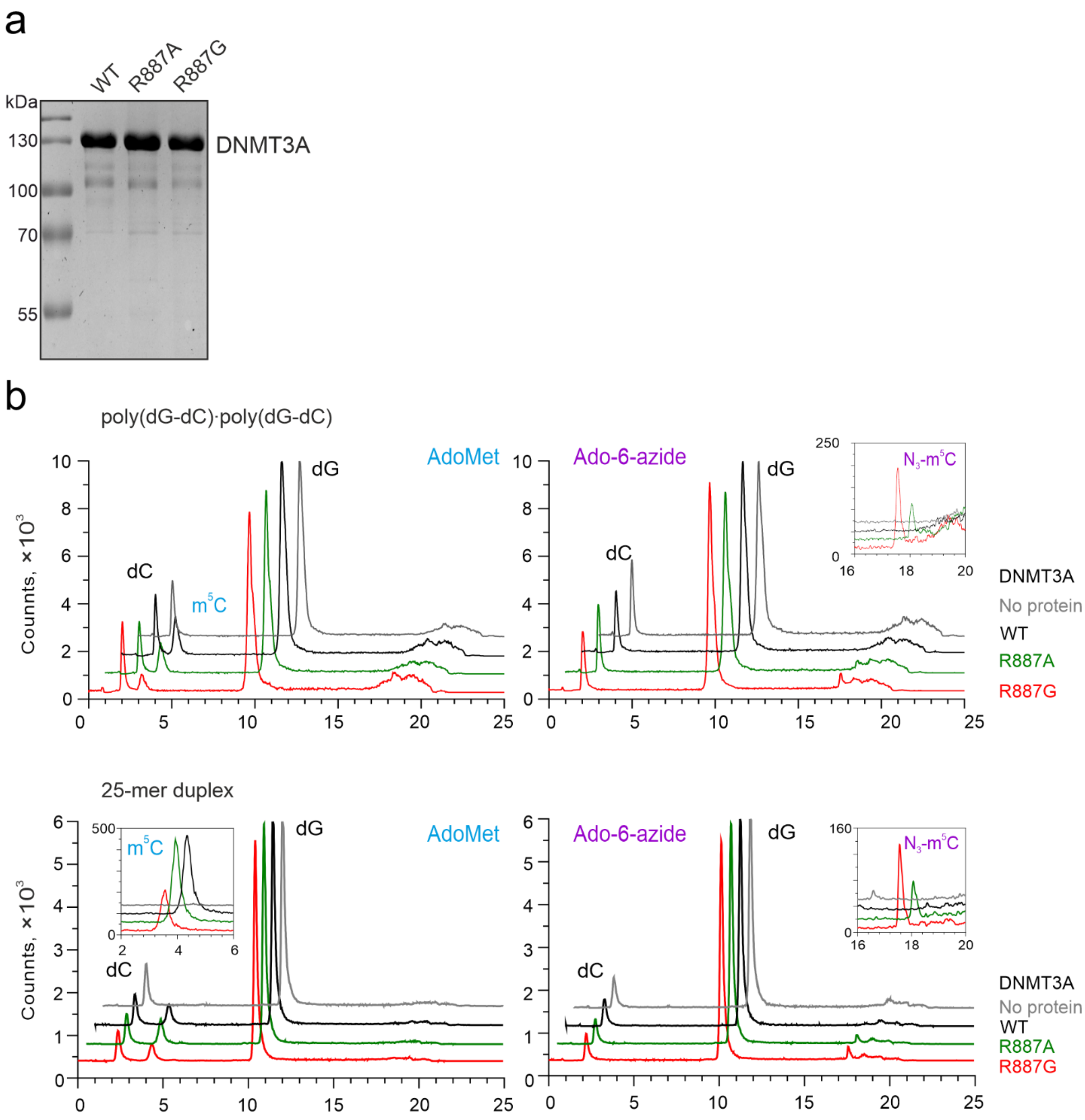

Figure S1. a) Recombinant DNMT3A protein variants with alanine or glycine substitutions at designated R887, respectively, were expressed in P. pastoris and purified using Ni^{2+} affinity chromatography. **b)** HPLC-MS/MS analysis of cytosine modifications by the DNMT3A variants in the presence of AdoMet or Ado-6-azide under steady-state conditions. Modification experiments were conducted with 6 µM poly(dG-dC)·poly(dG-dC) and unmethylated 25-mer oligonucleotide duplex DNA substrates, 100 µM AdoMet (left panel) or Ado-6-azide (right panel) and 800 nM of DNMT3A variants for 4 h at 37 °C. Inset boxes illustrate magnified sections around the modified cytosine nucleosides. m^5C, 5-methyl-2'-deoxycytidine; N_3-m^5C, 5-(6-azidohex-2-ynyl)-2'-deoxycytidine.

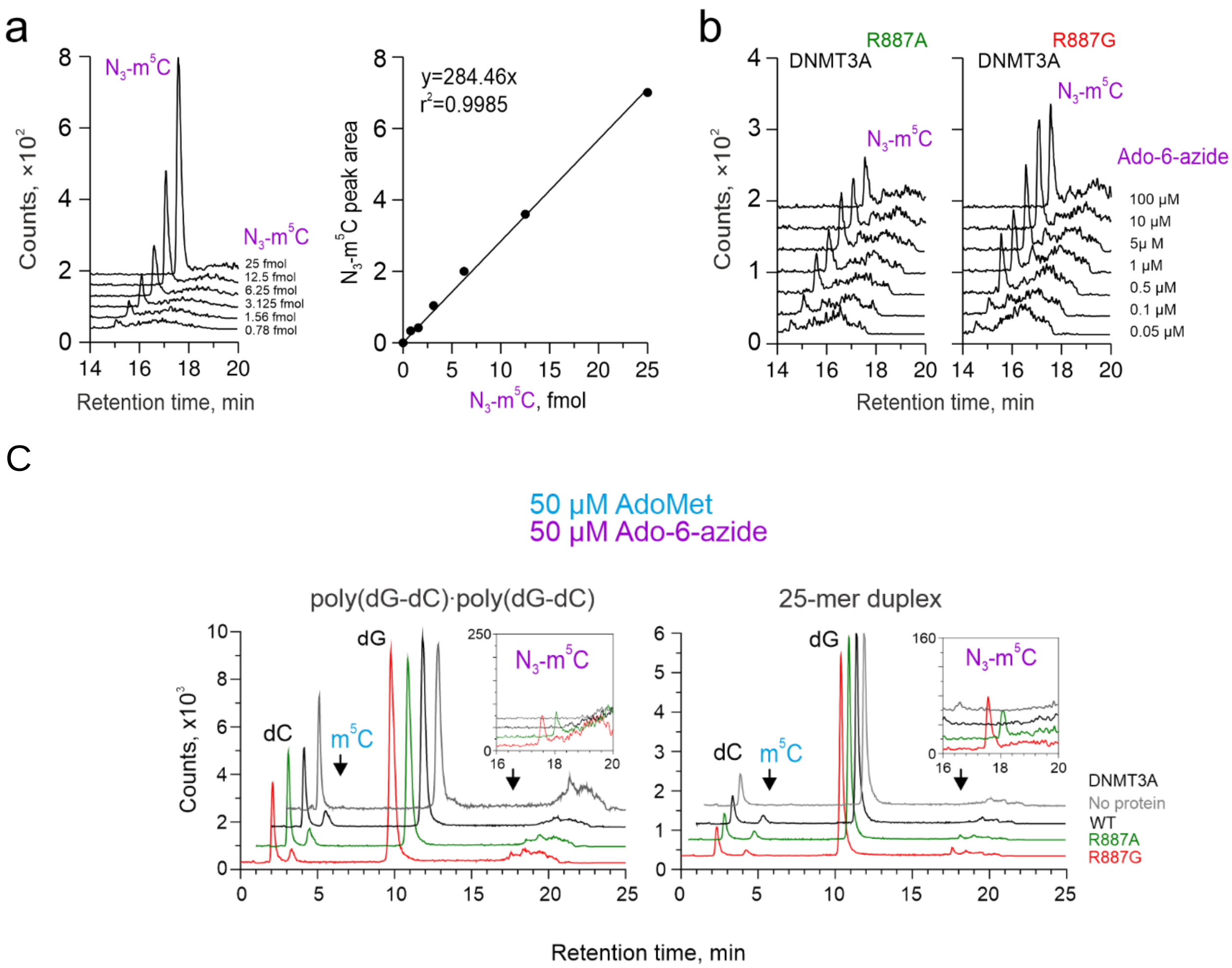

Figure S2. HPLC-MS/MS analysis of DNA modification by DNMT3A variants. a) Analysis of of standard azide-tagged nucleoside to determine correlation (calibration curve) between the amount analyzed and the chromatogram peak area enabling quantitative assessment of DNMT3A-specific labeling in genomic DNA. **b**) Representative HPLC-MS/MS analysis of azide-tagged cytosine nucleoside derived from DNMT3A-modified poly(dI-dC)·poly(dI-dC) DNA substrate in reactions containing increasing concentrations of Ado-6-azide cofactor. **c**) DNMT3A-specific DNA modification in the presence of AdoMet. DNA modification reactions were conducted using 6 µM of poly(dG-dC)·poly(dG-dC) or respective unmethylated 25-mer oligonucleotide duplex, 800 nM DNMT3A variants and 100 µM of cofactor in total. All reactions were carried out for 4 h at 37 ºC. Boxes show magnified sections of chromatographic N_3-m^5C peaks.

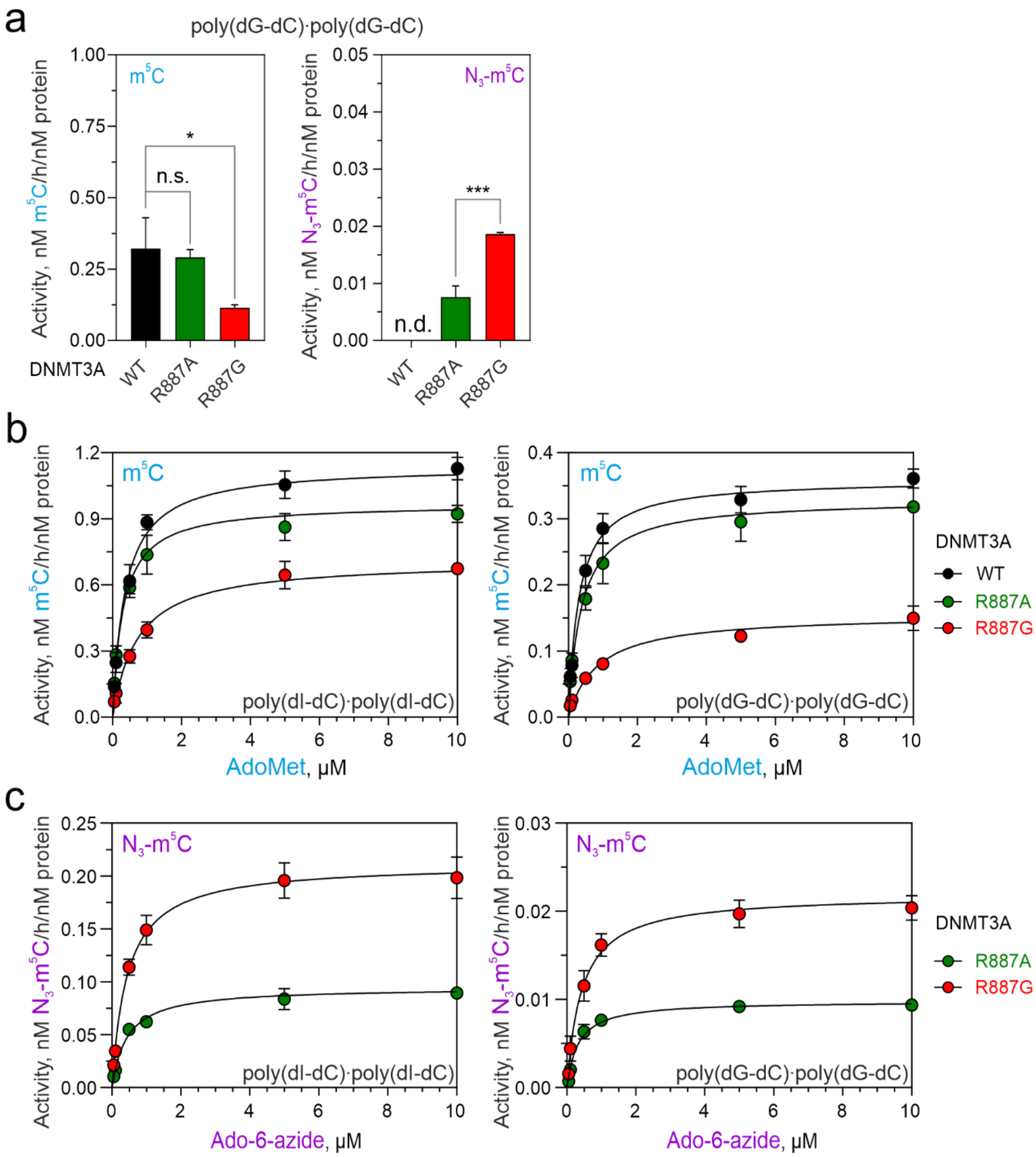

Figure S3. a) Catalytic activity of DNMT3A variants using poly(dG-dC)·poly(dG-dC) copolymer DNA. All experiments were performed using 6 µM of DNA substrate, 50 nM of each engineered protein and 100 µM cofactor incubating the reaction mixtures for 2 h at 37°C. Error bars denote ±SD. n.d. indicates not detected; n.s. not significant ($p > 0.05$); * $p < 0.05$; *** $p < 0.001$. Dependence of the DNMT3A modification activity on **b)** AdoMet or **c)** Ado-6-azide concentration across copolymer DNA substrates. The reactions were conducted with 0.02–10 µM AdoMet or Ado-6-azide, 6 µM (poly(dI-dC)·poly(dI-dC or poly(dG-dC)·poly(dG-dC)) DNA and 50 nM of each DNMT3A variant for 2 h at 37 °C, followed by HPLC-MS/MS analysis to assess the levels of DNA modification.

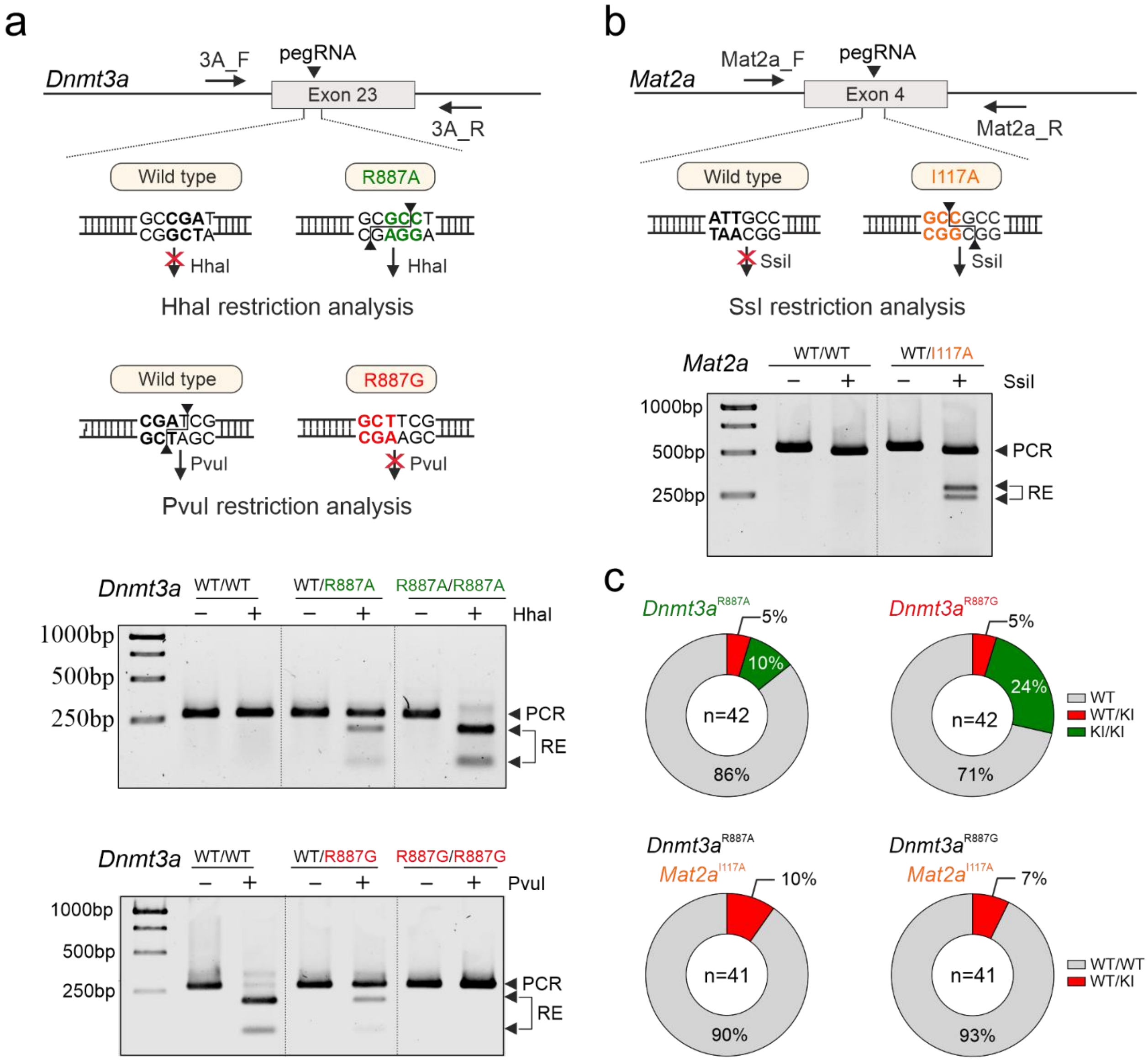

Figure S4. Derivation of monoallelic *Dnmt3a* R887A and R887G, *Mat2a* I117A knock-in mouse E14tg2a embryonic stem cells using genome prime editing. a) A schematic representation of the genome prime editing approach for the introduction of *Dnmt3a* R887A and R887G knock-in substitutions. Diagnostic restriction examination of PCR products in agarose gels (HhaI for *Dnmt3a* R887A, PvuI for R887G substitution) demonstrates effective heterozygous or homozygous prime editing of selected *Dnmt3a* codons in mESCs. **b)** Representative SsiI restriction analysis of PCR products indicates a heterozygous *Mat2a* I117A knock-in substitution in mESCs. **c)** PE knock-in efficiency of designated *Dnmt3a* and *Mat2a* codons in mESCs. n denotes a number of clones tested.

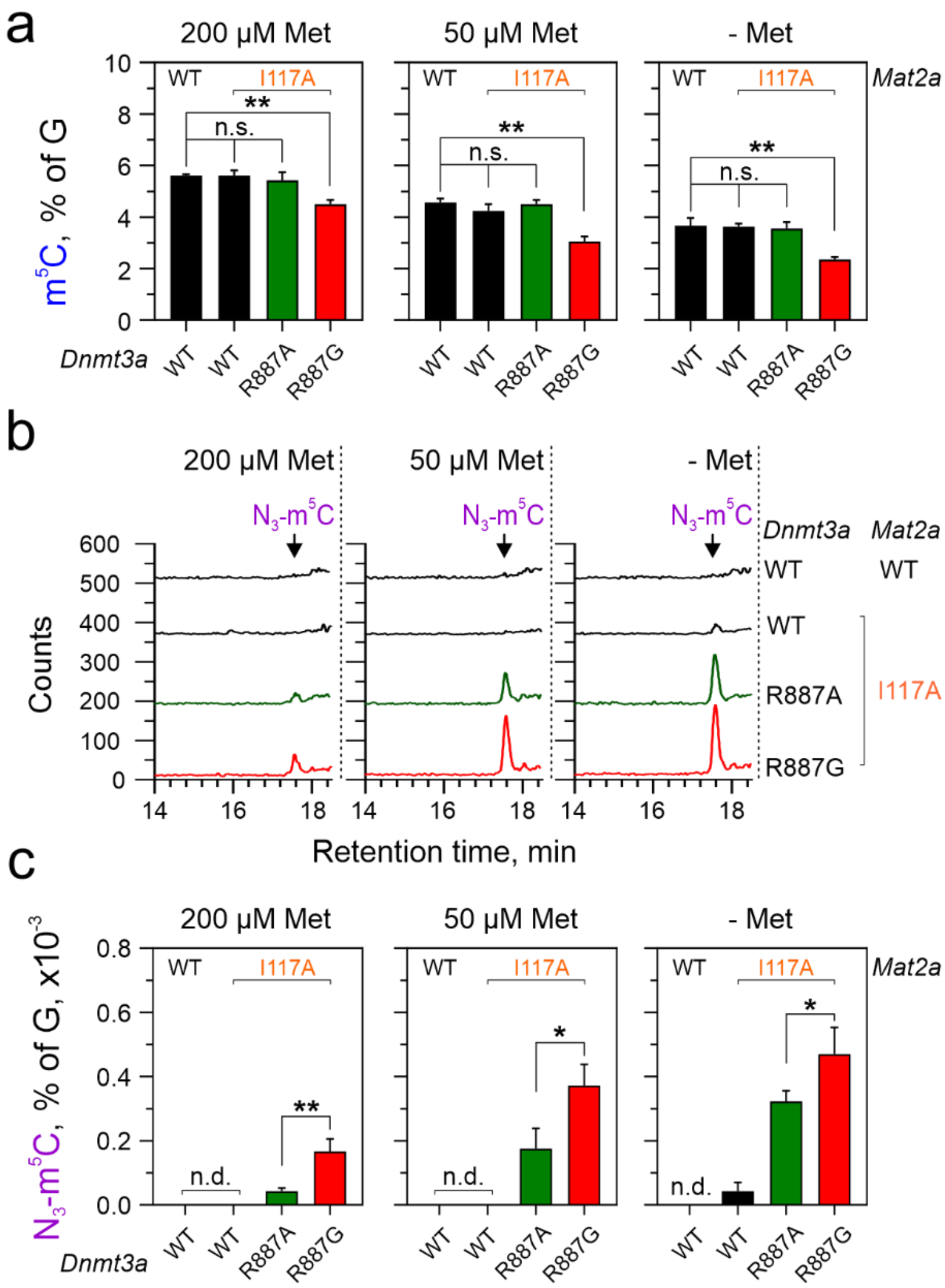

Figure S5. a) HPLC-MS/MS analysis of global m^5C levels in engineered mESCs in the presence of 200 µM, 50 µM or a total depletion of Met in cell culture medium. Bars represent mean ±SD, n=3 independent experiments. **b)** Representative HPLC-MS/MS chromatograms of genomic cytosine modification (N_3-m^5C) in mESC lines encoding engineered *Dnmt3a* and *Mat2a* variants after 24 h incubation with 1 mM N_3-Met in the presence or absence of Met. **c)** DNA azide-tagging levels in the engineered mESC lines post-metabolic genome labeling. Bars denote the mean expressed as % of dG ±SD, n=3 independent experiments; n.d. – not detected.

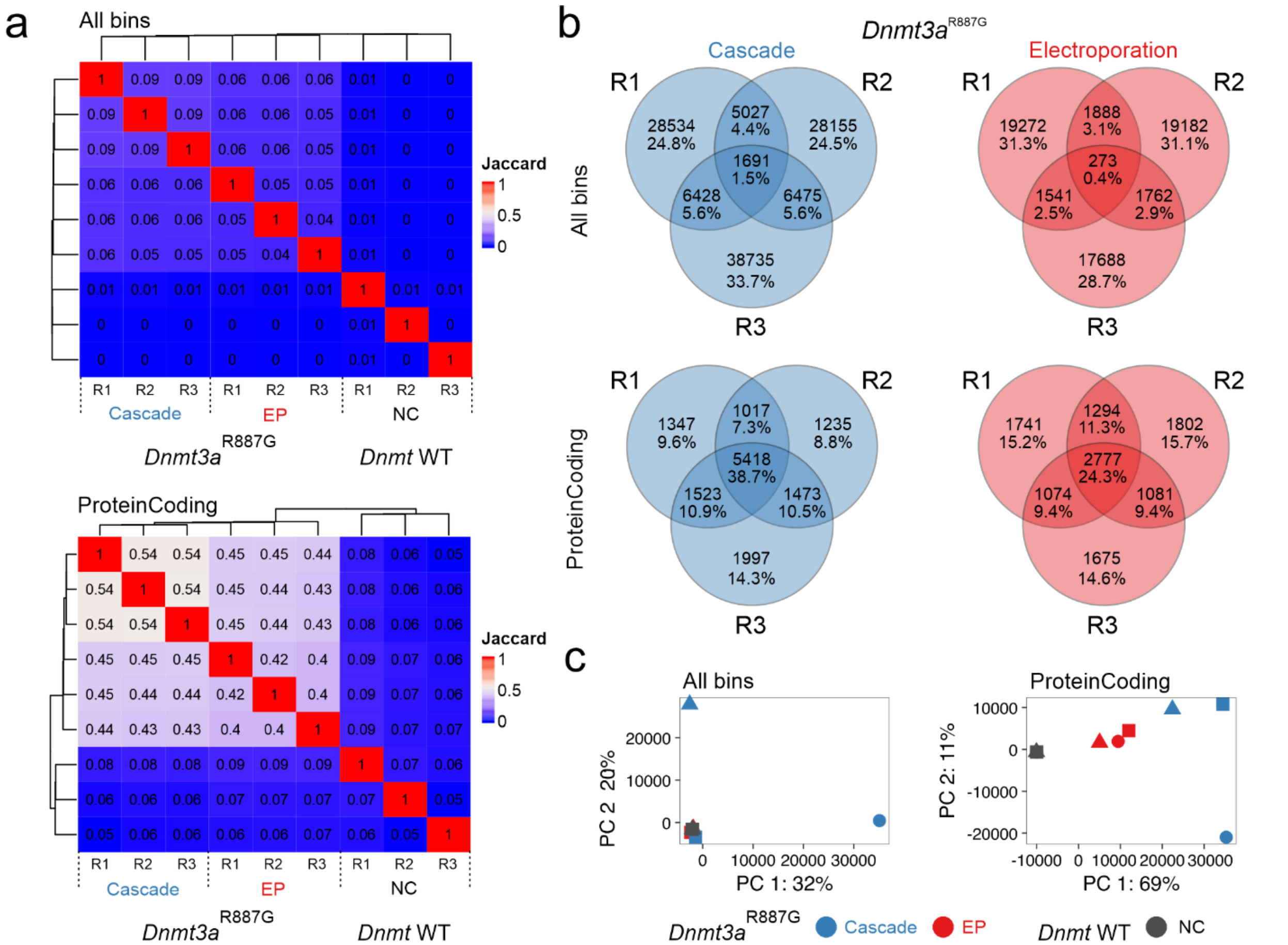

Figure S6. Statistical analysis of biological replicates of DNMT3A-TOP-seq datasets at the whole-genome and protein-coding regions. a) Jaccard similarity analysis of modified genomic bins between biological replicates (R1, R2, R3) of DNMT3A-tagged samples generated by electroporation with Ado-6-azide (EP) or metabolic labeling (Cascade) and negative controls (NC); b) Venn diagrams showing overlap of modified genomic bins between biological replicates; c) Principal component analysis of modified genomic bins.

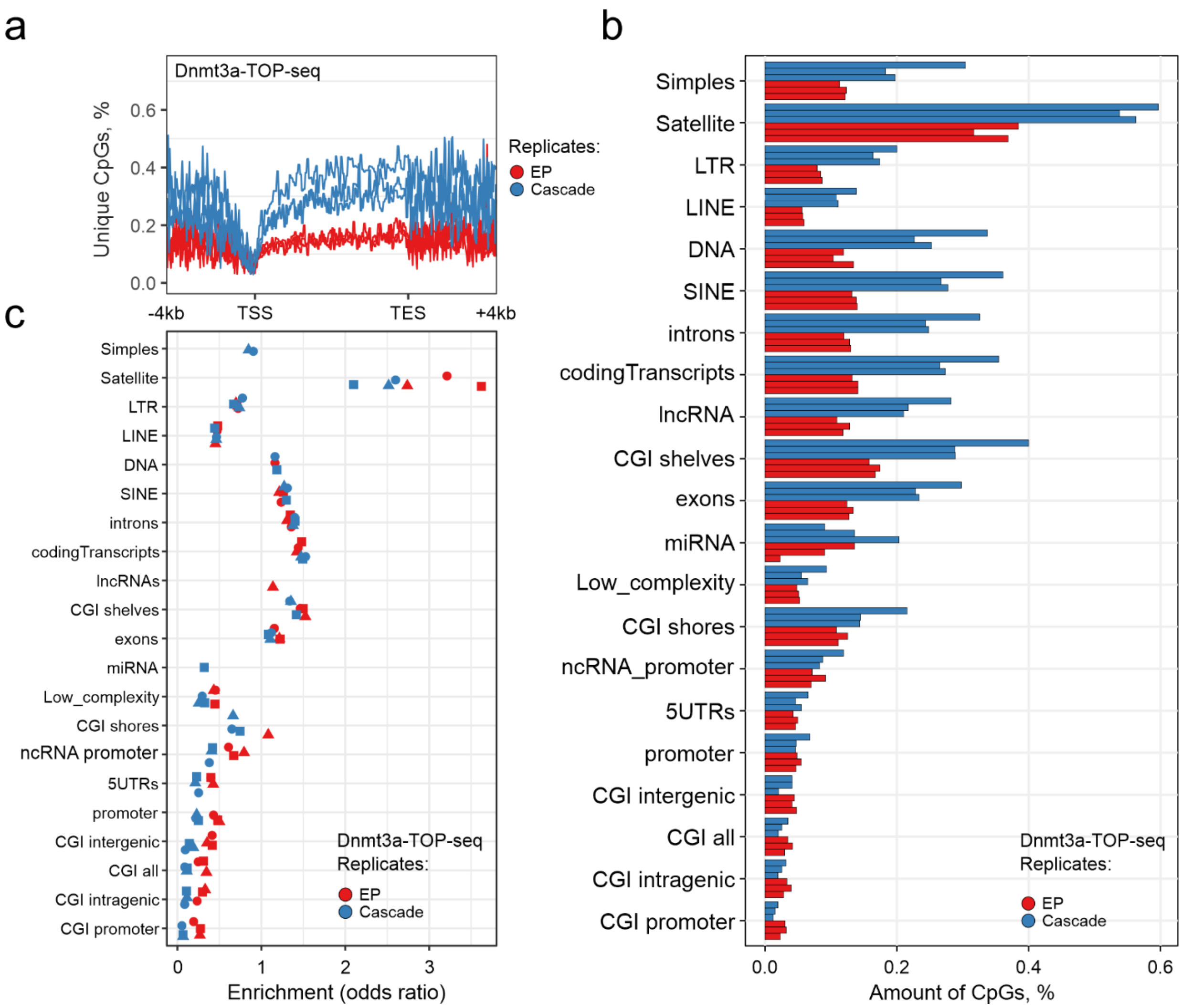

Figure S7. Analysis of DNMT3A-specific genome modification profiles using individual replicates of Dnmt-TOP-seq. a) Distribution of CpG modification along coding genes, including 4 kb flanking regions. **b)** Comparative enrichment analysis of genomic attributes. The odds ratio indicates whether a particular genomic element is enriched (>1) or reduced (<1) in the Dnmt-TOP-seq, WGBS (*Dnmt* WT) and TAB-seq (*Dnmt* WT) datasets. **c)** Comparative distribution of unique CpGs modified by DNMT3A across genomic elements and regulatory features.

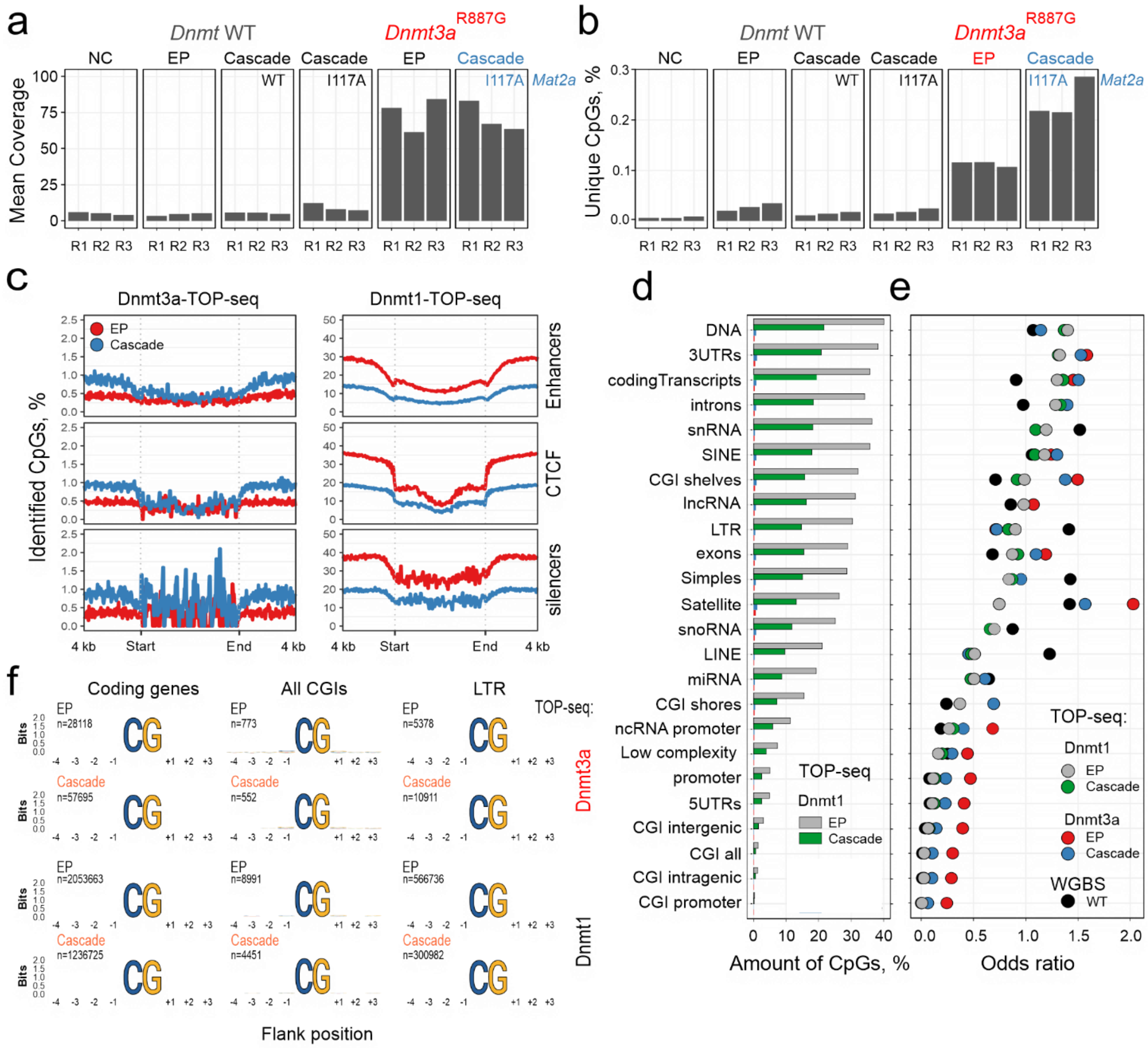

Figure S8. a) Average read coverage for identified CpGs in Dnmt3a-TOP-seq replicates. **b)** Percentage of unique CpGs in Dnmt3a-TOP-seq replicates. **c)** Distribution patterns of CpG modification across different genomic sites of enhancer, CTCF, and silencer regions in *Dnmt3a*^{R891G} and *Dnmt1*^{N1580A} knock-in cell Dnmt-TOP-seq datasets. **d)** Comparative distribution of unique CpGs modified by DNMT1 or DNMT3A across genomic elements and regulatory features. **e)** Comparative enrichment analysis of genomic attributes. The odds ratio indicates whether a particular genomic element is enriched (>1) or reduced (<1) in the Dnmt-TOP-seq datasets. **f)** DNMT3A or DNMT1-specific modification preferences at -4 to +3 flanks of CpG sequences in live mESCs. Profiles illustrate sequence preference in coding genes, CGI and LTR regions. n denotes a number of unique CpGs in Dnmt-TOP-seq datasets.

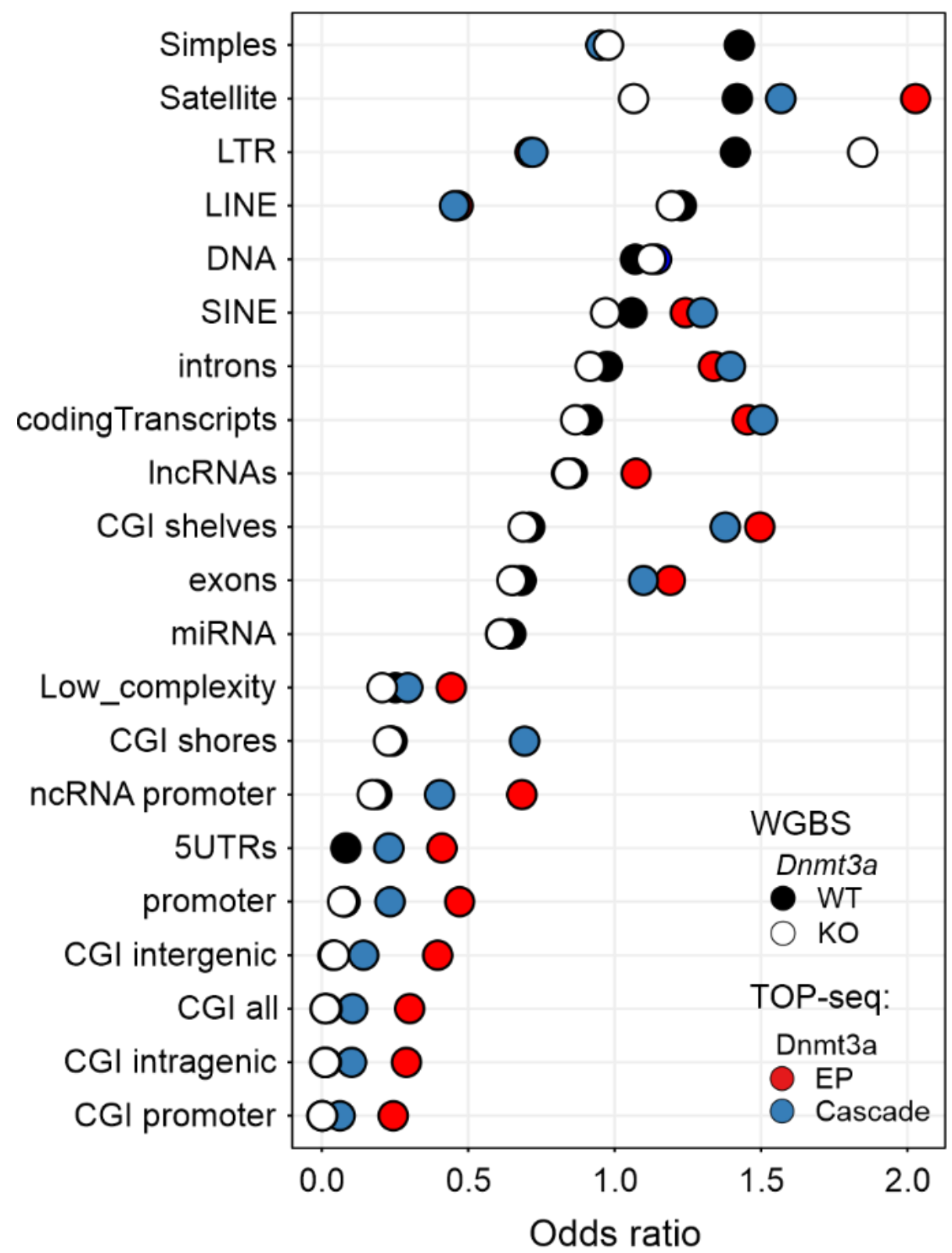

Figure S9. Comparative enrichment analysis of genomic attributes. The odds ratio indicates whether a particular genomic element is enriched (>1) or reduced (<1) in the Dnmt3a Dnmt-TOP-seq and *Dnmt3a* KO WGBS (retrieved from GSE100957) datasets.

Supplementary Tables S1-S4

Table S1. Oligonucleotides used for Dnmt3A prime editing.

Scaffold_Fw	5'-AGAGCTAGAAATAGCAAGTTAAAATAAGGCTA GTCCGTTATCAACTTGAAAAAGTGGCACCGAGTCG
Scaffold_Rv	5'-GCACCGACTCGGTGCCACTTTTTCAAGTTGATAAC GGACTAGCCTTATTTTAACTTGCTATTTCTAG
pegRNA Spacer Fw	5'-CACCTGACCGGCACGCTCCACGATGTTTT
pegRNA Spacer Rv	5'-CTCTAAAACATCGTGGAGCGTGCCGGTCA
R887G pegRNA Fw	5'-GTGCCTGCTGGGCGGCTCGTGGAGCGTGCCG
R887G pegRNA Rv	5'-AAAACGGCACGCTCCACGAGCCGCCCAGCAG
R887A pegRNA Fw	5'- GTGCCTGCTGGGCGCCTCGTGGAGCGTGCCG
R887A pegRNA Rv	5'- AAAACGGCACGCTCCACGAGGCGCCCAGCAG
sgRNA Fw	5'-CACCGCTTGTGTGTAAGGGACATGG
sgRNA Rv	5'-AAACCCATGTCCCTTACACACAAGC
Dnmt3a_PCR Fw	5'-GGGTGTTTGGCTTCCCCGT

Dnmt3a_PCR Rv	5'-CCTTTTTCTCTTCTGGGTGCTGAAC

Table S2. Oligonucleotides used for Dnmt1-TOP library preparation.

A1_adapter	5'-P-GATTGGAAGAGTGGTTCAGCAGGAATGCTGAG
A2_adapter	5'-ACACTCTTTCCCTACATGACACTCTTCCAATCT
Biotinylated alkyne-containing DNA oligonucleotide	5'-T**X**TTTTGTGTGGTTTGGAGACTGACTACCAGATGTAACA-Biotin; X=C8-Alkyne-dT
Complementary priming strand	5'-TGTTACATCTGGTAGTCAGTCTCCAAACCACACAA
A(Ad)-EP-barcodeA1-primer	5′-CCATCTCATCCCTGCGTGTCTCCGACTCAGCTAA GGTAACTGTTACATCTGGTAGTCAGTCTC
A(Ad)-EP-barcodeA6-primer	5′-CCATCTCATCCCTGCGTGTCTCCGACTCAGTAAGG AGAACTGTTACATCTGGTAGTCAGTCTC
Ad(trP1)-A2-primer	5’-CCTCTCTATGGGCAGTCGGTGATCACTCTTTCCCTACATGACACT

Table S3. Dnmt-TOP-seq sequencing statistics

Sample ID	Raw reads	Processed Reads	Aligned reads	Identified CpGs	Mean coverage
NC R1	451199	122557	8063	1320	6.1
NC R2	363419	111161	7083	1295	5.5
NC R3	399429	122454	8219	1946	4.2
WT EP R1	215351	84679	15110	4406	3.4
WT EP R2	435296	149542	28717	5992	4.8
WT EP R3	630787	211820	41645	7686	5.4
WT cascade R1	704811	219318	14134	2450	5.8
WT cascade R2	977700	275014	18178	3176	5.7
WT cascade R3	1198083	294752	19087	3921	4.9
I117A cascade R1	1172786	296124	39961	3217	12.4
I117A cascade R2	759331	205925	31758	3906	8.1
I117A cascade R3	1216776	278157	40349	5442	7.4
Dnmt3a R887G EP_R1	115791288	9216807	1984960	25333	78.4
Dnmt3a R887G EP_R1	106517386	8474803	1566019	25447	61.5
Dnmt3a R887G EP_R1	125999255	9769643	1977942	23419	84.5
Dnmt3a R887G Cascade_R1	113410068	9327270	3971462	47688	83.3
Dnmt3a R887G Cascade_R2	111783259	9216219	3167088	47085	67.3
Dnmt3a R887G Cascade_R3	98199389	11272239	3979405	62508	63.7

Table S4. Raw sequencing statistics

GEO series ID	GEO sample ID	NCBI BioProject ID	NCBI BioSample ID	NCBI SRA ID	NCBI GEO Sample title	Sample	Raw reads	Raw size, Gb	Average read length	Max read length	Average read quality
GSE 297788	GSM8999562	PRJNA 1266178	SAMN48677128	SRX28890344	Wild type DNMT3A, MAT2A I117A rep1	I117A cascade	1172786	0.14	198	352	23.8
	GSM8999563		SAMN48677127	SRX28890345	Wild type DNMT3A, MAT2A I117A rep2	I117A cascade	759331	0.18	183	374	24.1
	GSM8999564		SAMN48677126	SRX28890346	Wild type DNMT3A, MAT2A I117A rep3	I117A cascade	1216776	0.21	173	376	23.9
	GSM8999565		SAMN48677125	SRX28890347	Wild type DNMT3A rep1	WT EP	215351	0.04	180	352	23.7
	GSM8999566		SAMN48677124	SRX28890348	Wild type DNMT3A rep2	WT EP	435296	0.08	184	350	23.9
	GSM8999567		SAMN48677123	SRX28890349	Wild type DNMT3A rep3	WT EP	630787	0.11	171	382	23.6
	GSM8999568		SAMN48677122	SRX28890350	Wild type DNMT3A NC rep1	NC	451199	0.09	197	354	23.5
	GSM8999569		SAMN48677121	SRX28890351	Wild type DNMT3A NC rep2	NC	363419	0.07	184	371	23.7
	GSM8999570		SAMN48677120	SRX28890352	Wild type DNMT3A NC rep3	NC	399429	0.07	169	367	23.7
	GSM8999571		SAMN48677119	SRX28890353	DNMT3A R887G rep1	Dnmt3a R887G EP	115791288	17.47	150	393	23.8
	GSM8999572		SAMN48677118	SRX28890354	DNMT3A R887G rep2	Dnmt3a R887G EP	106517386	15.82	148	391	23.8
	GSM8999573		SAMN48677117	SRX28890355	DNMT3A R887G rep3	Dnmt3a R887G EP	125999255	18.55	147	391	23.5
	GSM8999574		SAMN48677116	SRX28890356	DNMT3A R887G, MAT2A I117A rep1	Dnmt3a R887G cascade	113410068	16.9	149	393	23.3
	GSM8999575		SAMN48677115	SRX28890357	DNMT3A R887G, MAT2A I117A rep2	Dnmt3a R887G cascade	111783259	15.7	140	388	23.4
	GSM8999576		SAMN48677114	SRX28890358	DNMT3A R887G, MAT2A I117A rep3	Dnmt3a R887G cascade	98199389	14.36	146	390	23.3
